# Decoding leukemia at the single-cell level: clonal architecture, classification, microenvironment, and drug resistance

**DOI:** 10.1186/s40164-024-00479-6

**Published:** 2024-01-30

**Authors:** Jianche Liu, Penglei Jiang, Zezhen Lu, Zebin Yu, Pengxu Qian

**Affiliations:** 1 Center for Stem Cell and Regenerative Medicine and Bone Marrow Transplantation Center of the First Affiliated Hospital, School of Medicine, Zhejiang University, Hangzhou, 310058 China; 2 Liangzhu Laboratory, Zhejiang University, 1369 West Wenyi Road, Hangzhou, 311121 China; 3 Institute of Hematology, Zhejiang Engineering Laboratory for Stem Cell and Immunotherapy, Zhejiang University, Hangzhou, 310058 China; 4 International Campus, Zhejiang University-University of Edinburgh Institute (ZJU-UoE Institute), Zhejiang University School of Medicine, Zhejiang University, 718 East Haizhou Road, Haining, 314400 China

**Keywords:** Leukemia, Single-cell sequencing, Tumor microenvironment, Drug resistance

## Abstract

Leukemias are refractory hematological malignancies, characterized by marked intrinsic heterogeneity which poses significant obstacles to effective treatment. However, traditional bulk sequencing techniques have not been able to effectively unravel the heterogeneity among individual tumor cells. With the emergence of single-cell sequencing technology, it has bestowed upon us an unprecedented resolution to comprehend the mechanisms underlying leukemogenesis and drug resistance across various levels, including the genome, epigenome, transcriptome and proteome. Here, we provide an overview of the currently prevalent single-cell sequencing technologies and a detailed summary of single-cell studies conducted on leukemia, with a specific focus on four key aspects: (1) leukemia’s clonal architecture, (2) frameworks to determine leukemia subtypes, (3) tumor microenvironment (TME) and (4) the drug-resistant mechanisms of leukemia. This review provides a comprehensive summary of current single-cell studies on leukemia and highlights the markers and mechanisms that show promising clinical implications for the diagnosis and treatment of leukemia.

**Supplementary Information:**

The online version contains supplementary material available at 10.1186/s40164-024-00479-6.

## Introduction

Leukemias are lethal blood malignancies that are characterized by abnormal clonal proliferation of hematopoietic cells [1]. Due to the malignant transformation of hematopoietic stem/progenitor cells induced by mutation, their normal hematopoietic function is damaged, resulting in uncontrolled proliferation, dysregulated differentiation, and impaired apoptosis.

**Fig. 1:**
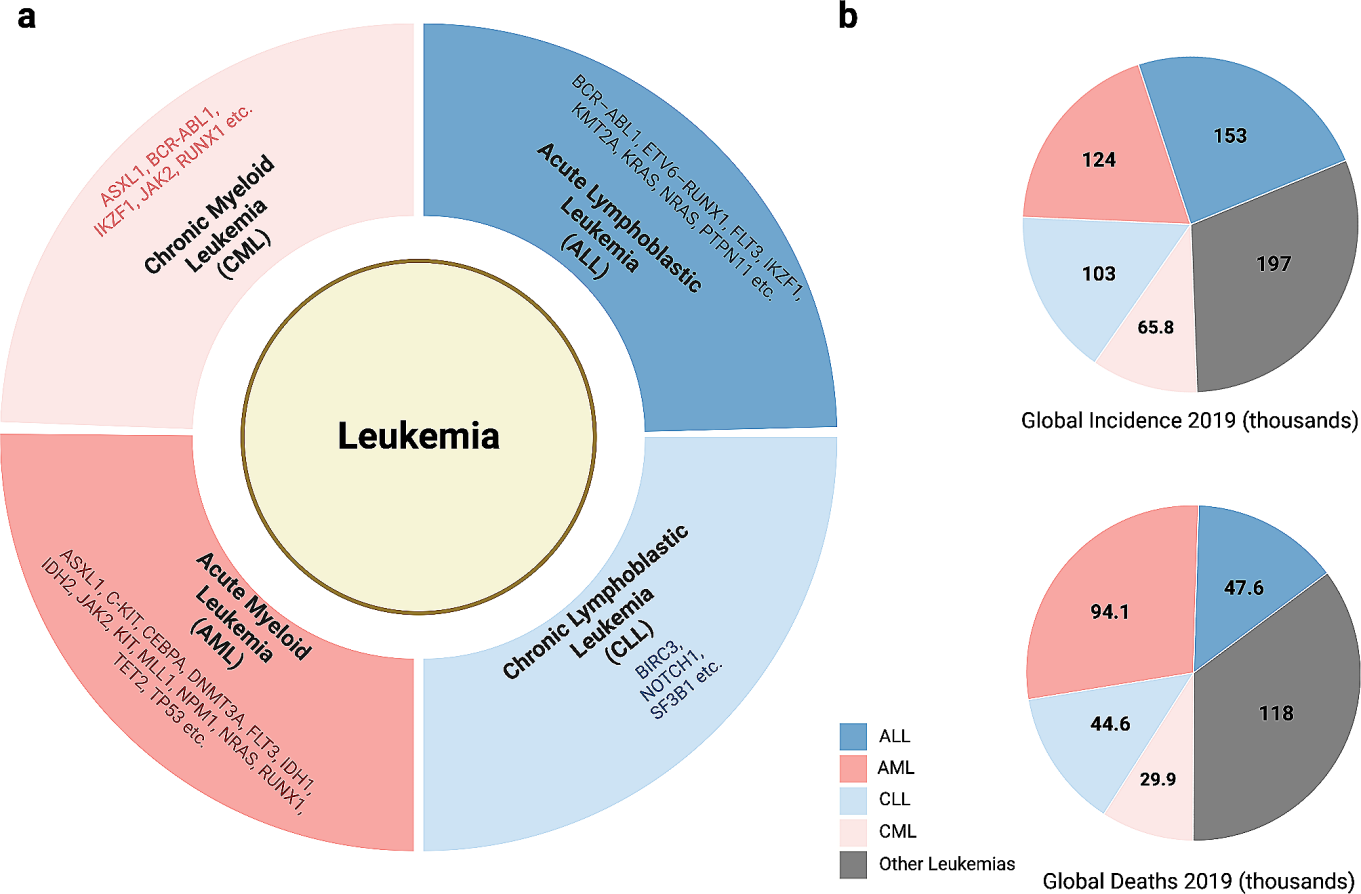
Summary of major leukemia subtypes. (**a**) Summary of the most prevailing oncogenetic mutation for the four main subtypes of leukemia. (**b**) Visualization of the global incidence and death of different leukemia types in 2019. Data was retrieved from the 2019 Global Burden of Disease (GBD) study [2]

Leukemias are clinically subcategorized according to morphology, immunophenotype, cytogenetic and molecular features [1], including acute myeloid leukemia (AML), acute lymphoblastic leukemia (ALL), chronic myeloid leukemia (CML) and chronic lymphoblastic leukemia (CLL) (Fig. 1a). According to the latest Global Burden of Disease (GBD) study in 2019 [2], AML had the highest death rate among the main types of leukemia while ALL showed the highest increase in occurrence (Fig. 1b). Also, AML is the most severe leukemia type and prevails most in adults [3], while ALL occurs most frequently in children [4]. Over the last decade, there has been an unparalleled expansion of treatment options for leukemia, including novel chemotherapy regimens, monoclonal antibodies, small molecule inhibitors, and Chimeric antigen receptor T cell (CAR-T) therapy [5–8]. This was accompanied by an ever-growing comprehension of leukemia pathogenesis. However, the outcomes for AML and adult ALL remain unsatisfactory, and relapse of leukemias poses a significant challenge [6, 9]. This is partly due to the immense heterogeneity in different subtypes of leukemia, making it challenging to develop targeting drugs specifically. To gain a better understanding of the pathogenic mechanisms involved, comprehensive analyses of the entire genome have been utilized to identify heterogeneous molecular traits. However, the drawback of the bulk analysis approach is that while it offers an overall profile, it may mask the traits displayed by a specific subset of cells.

Recently, the transformative rise of single-cell sequencing technology offered an unprecedently high resolution for interrogation on a single cell. Compared to bulk sequencing, single-cell sequencing provides an exclusive advantage in identifying cell-specific information. In addition to profiling single cells, it also has the innate ability to decipher cell-cell interaction networks in intricate cell systems [10] and can reconstruct the phylogenetic trajectory to better organize the clonal architecture in tumors [11]. For example, by inferring ligand-receptor activity specific to two cell types, single-cell transcriptome profiling revealed distinct communication statuses in multiple tumor niches that are masked by bulk methods [12–14]. Also, in terms of profiling the intra-tumor identity in subclones, traditional bulk methods assume mutations arise from the same subclone if they have similar mutant allele frequencies [15], which poses an immense drawback in that it is difficult to distinguish subclones if they have similar mutant allele frequencies. However, single-cell sequencing overcomes this issue by directly looking into the mutational landscape in single cells and it has seen a large application of incorporating single-cell genomics to group the clonal architecture in different cancers [16].

Single-cell technology is extremely helpful in characterizing genetic and epigenetic regulation, transcriptional, translational, and post-translational heterogeneity within a cell, and allows the integration of multi-omics level interaction networks, opening the new world for leukemia characterization [17–20]. It was widely acknowledged that leukemia is a highly heterogeneous malignancy [1], exhibiting significant differences in (1) the mutations and regulatory elements involved in tumor evolution, (2) the surface biomarkers used to subtype leukemia and predict prognosis, (3) the tumor microenvironment that may underlie its pathogenesis and relapse, and (4) the mechanisms that confer resistance to drugs and relapse. Single-cell studies have been extensively conducted to determine the clonal architecture, subtyping leukemia, characterizing the tumor microenvironment, and revealing drug response and resistance. Prospects of these single-cell studies have facilitated the precise diagnosis, innovation of targeted therapy, and prognosis prediction in leukemia. Here, we reviewed the application of single-cell sequencing technology in leukemia, with a focus on the advances in AML, CML, ALL, and CLL. We mainly summarized these studies into (1) disclosure of clonal evolution, (2) determining leukemia subtypes, (3) characterizing the tumor microenvironment, and (4) revealing drug-resistant mechanisms.

## Single-cell omics

**Fig. 2:**
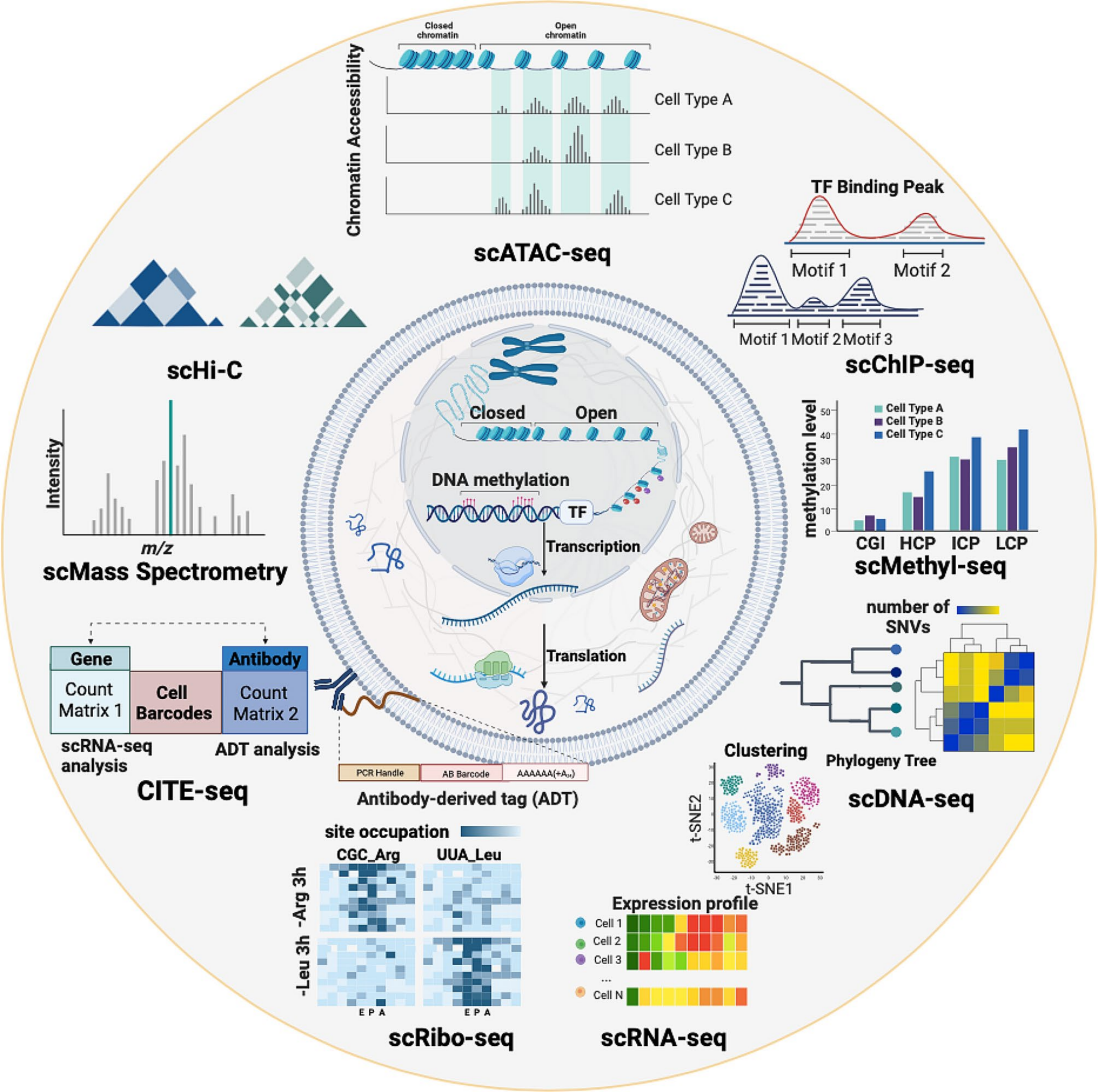
Summary of single-cell technologies. Single-cell technologies are able to dissect multi-layered cell information lining the central dogma, ranging from genomic, and epigenomic, to transcriptomic, proteomics, and even tranlatomics. scDNA-seq allowed variant detection, and phylogeny construction, whereas scRNA-seq unravels heterogeneity based on expression dynamics. scHi-C, sc-ATAC-seq and scChIP-seq, and scMethyl-seq separately disclosed cell-specific epigenetic regulation by layers of 3D chromosomal conformation, chromosomal accessibility, DNA-binding protein and histone modifications, and DNA methylation. scMass-spectrometry allowed the dissection of the functional unit, linking direct phenotype, and scRibo-seq allowed translational profiling. Multi-omics approaches like CITE-seq allowed simultaneous profiling of transcriptomics and epitope information

Single-cell sequencing technology has rolled its wheel with increasingly rapid speed since 2009, when Tang et al. first described single-cell RNA sequencing (scRNA-seq [21]). Since then, multi-layered single-cell dissection techniques have emerged to characterize the cells thoroughly (Fig. 2). At the genomic level, methodologies such as single-cell DNA sequencing (scDNA-seq [22]) and single-cell whole exome sequencing (scWGS-seq [23]) enable the exhaustive examination of mutations and copy number alterations within single cells. Deeper into the regulatory complexities, methodologies were developed for the investigation of single-cell epigenetic landscapes. For example, single-cell Hi-C (scHi-C [24]) could provide insights into the higher-order chromosomal structure and elucidate the spatial organization of the genome, single-cell bisulfite sequencing (scBS-seq, or scMethyl-seq [25]) focuses on the detecting methylation modification of DNA and thus unravel the methylation heterogeneity in single-cells. Besides these, single-cell Assay for Transposase-Accessible Chromatin Sequencing (scATAC-seq [26]) and single-cell Chromatin Immunoprecipitation Sequencing (scChIP-seq [27]) further intricacies of chromatin biology by delineating profiles of open chromatin regions and protein-DNA interactions, respectively.

While the regulatory landscape is explored at the genomic and epigenomic levels, single-cell RNA sequencing (scRNA-seq) continues to occupy a central role in advancing single-cell studies, and has gained the largest application. Its widespread adoption is attributed to a well-established experimental and computational pipeline and its unparalleled ability to link expressional heterogeneity across diverse cell populations. Simultaneously, the advent of single-cell Mass Spectrometry (scMass Spectrometry [28, 29]) has brought about another transformative dimension by allowing the direct association of molecular phenotype with protein expression at the single-cell level. Most recently, there have been notable advancements in characterizing the translatome through single-cell Ribosome sequencing (scRibo-seq [30]). By profiling the state of translational machines, and ribosomes, the technology is promising in adding an additional layer of information for exploring cell heterogeneity in terms of translational dynamics. These single-cell technologies are also summarized in Fig. 2.

As the diversity of single-cell technology proliferated, the simultaneous conduction of multiple omics at the single-cell level (single-cell multiomics) came to the front, enabling a more precise definition of cellular characterization and comprehensive exploration of transcriptional regulatory mechanisms [18]. For example, single-nucleus chromatin accessibility and mRNA expression sequencing (SNARE-seq) is a large-scale profiling method that simultaneously measures single-cell transcriptome with its chromatin-accessible region in one cell, enabling the elucidation of the chromatin accessibility landscape and its impact on transcription [31]. Single-cell triple omics sequencing technique (scTrio-seq) realized the tri-profiling of copy number variation (CNV), DNA methylome, and transcriptome in the same single cell, thus making the links and regulatory networks among these various layers [32]. The cellular indexing of transcriptomes and epitopes (CITE-seq [33]), utilizing oligonucleotide-labeled antibodies to link the surface protein with the cellular transcriptome, has recently gained large application in leukemia [34–38] with its ability to provide additional information on surface hallmarks of the cell and enabling the antigen-specific dissection of cancer.

## Single-cell analyses further reveal clonal evolutionary patterns and driver events in leukemia

**Fig. 3:**
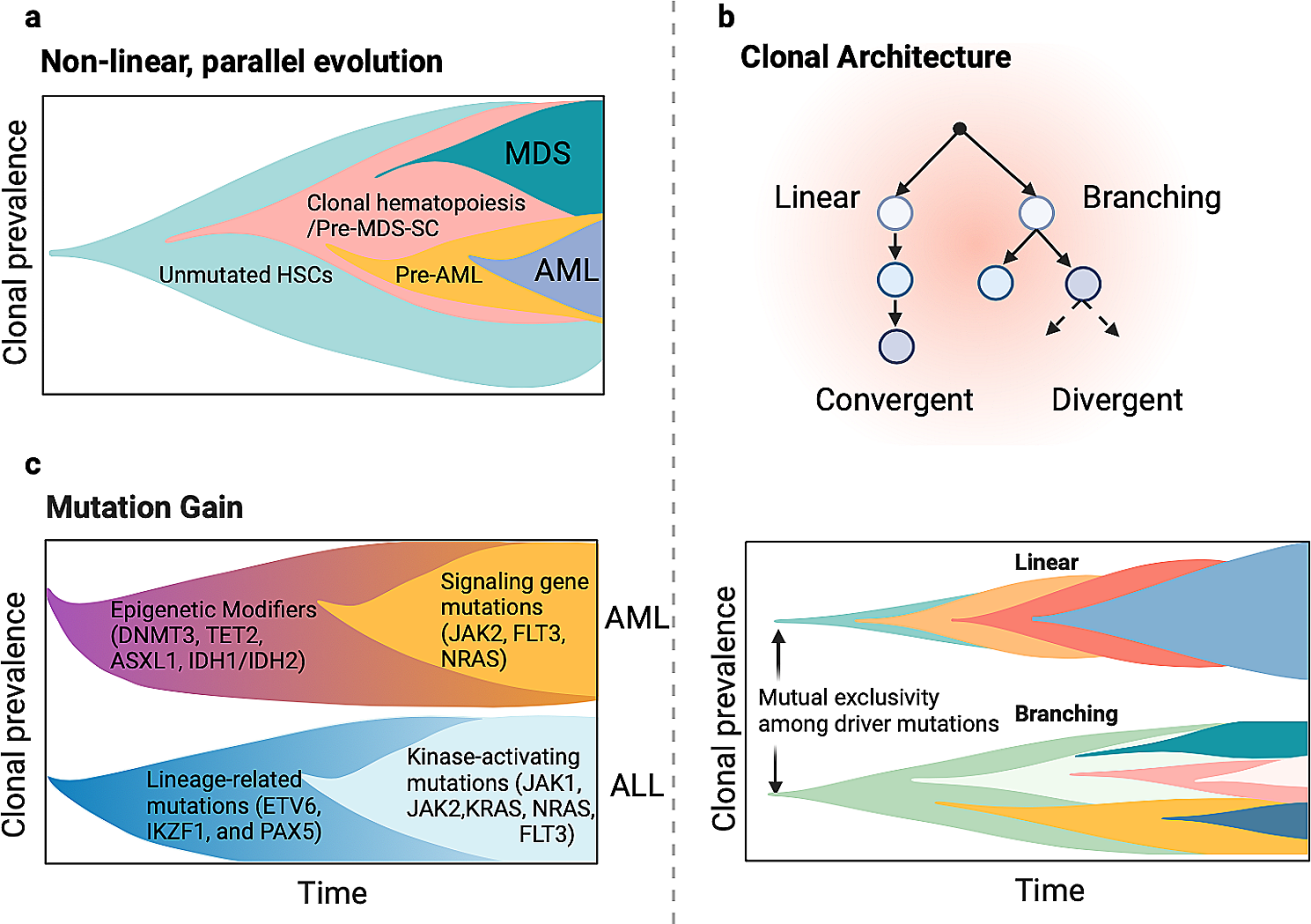
Single-cell sequencing reveals differential clonal evolutionary and mutational patterns in leukemia. (**a**) Non-linear, parallel clonal evolution was found from clonal hematopoiesis to MDS or AML [41]. (**b**) The typical AML leukemia evolutionary pattern is summarized. Mutual exclusive driver mutations are frequently observed in different subclones, where subsequent branched or linear clonal architecture was found [43]. (**c**) The sequence of mutation gain was also depicted by single-cell studies in AML and ALL, where DITA (DNMT3A, TET2, ASXL1 and/or IDH1 or IDH2) is the most prevalent initiating mutation in AML [15], and linage-related mutation often occurs earlier than kinase-activating mutations in ALL [49]

Clonal evolution is a landmark theory that attributes cancer pathogenesis to an evolutionary process driven by mutations and the selective advantages of subclones [39]. The identification of the pattern in cancer clonal evolution and the driver events that conferred selective advantages in tumor progression is of clinical importance. Applying single-cell sequencing techniques can dissect tumors at the cellular level, further revealing the pathogenesis and clonal evolution processes with ultra-high resolution. Here, we summarized the recent single-cell studies revealing leukemia clonal structure, sequential mutation gain and driver events (Fig. 3; Table 1).

**Table 1:** Summary of key findings related to clonal evolution in leukemia by single-cell sequencing

Leukemia Type	Major Methods	Key Findings	Clinical Relevance	References
MDS, AML	Single-cell targeted DNA sequencing	Pre-MDS stem cells and MDS stem cells contributed to MDS transformation to AML in a nonlinear and parallel clonal evolutionary pattern	Identified that crucial role of small and diverse aberrant stem cell subpopulations may confer leukemic transformation	[41]
CH, MPN, AML	scDNA-seq; Simultaneous single-cell mutational profiling and immunophenotyping.	Increased clonal complexity was observed from CH to MPN to AML. Mutations in signaling genes often occur in distinct subclones more than once. Noted that epigenetic modifiers such as DTAI (DNMT3A, TET2, ASXL1, and/or IDH1 or IDH2) are the most prevalent AML-initiating mutation and the combination of them may confer clonal dominance.	Identified multiple important characteristics in the clonal architecture along the progression of AML.	[15]
AML	scDNA-seq; Simultaneous single-cell mutational profiling and immunophenotyping.	Driver mutations of AML are often in a co-occurring and mutually exclusive pattern. Linear and branching pattern of AML phylogeny was observed, and some of the branching patterns showed convergence.	Summarized the mutational and phylogenetic features in AML that may underly risk stratification and prognosis determination.	[43]
CLL	scRNA-seq; single-cell targeted mutation analysis in DNA and RNA	LCP1 and WNK1 were identified as novel CLL drivers. Convergent expression profile was detected in CLL despite genetic differences.	Identified novel driver mutation for therapeutic targeting CLL. Highlighted that the targeted scRNA mutation analysis may sensitively determine the mutation profile with transcriptomics.	[44]
ALL	Targeted scDNA-seq of SNVs, deletions and lgH	Structural variants mostly occur before SNVs in ALL. KRAS occurs in late ALL development and is not enough to confer clonal dominance.	Ordered genetic events of ALL, which is prognostically informative. Characterized the function of KRAS mutation in ALL.	[46]
T-ALL	Targeted scDNA-seq; scRNA-seq	Mutation gain was ordered in T-ALL. Inactivation of CDKN2A/B and T-cell receptor deletions and fusion genes are intermediate events and NOTCH1 mutation is the late event.	Emphasized the importance of targeting NOTCH signaling in T-ALL.	[47]
T-ALL	Targeted scDNA-seq	NOTCH1 mutation can also be detected at diagnosis of the T-ALL although typically occurs later. The presence of small clones at diagnosis can evolve into major clones in later stages.	Revealed the heterogeneity of NOTCH1 mutation in different subpopulations and provided evidence for differentially targeting the NOTCH pathway.	[48]
ALL	scDNA-seq; Simultaneous targeted single-cell DNA sequencing and cell-surface protein expression analysis	Lineage related mutations (ETV6, IKZF1, and PAX5) occurs earlier than kinase activating mutations (JAK1, JAK2, KRAS, NRAS, FLT3)	Highlighted and summarized the sequential gain of the genetic event in ALL, which may be prognostically informative.	[49]
MPN, AML	scRNA-seq	Increased expression of DUSP6 underlies JAKi resistance disease transformation from MPN to sAML. DUSP6 functions through the DUSP6-RSK1-S6 axis. Pharmacological inhibition of DUSP6 eliminated the resistance to JAKi.	Highlighted DUSP6-RSK1 is a vulnerable, therapeutically targetable pathway in myeloid malignancies.	[51]
MPN, AML	TARGET-seq	The effect of chronic inflammation in TP53-mutation-driven clonal evolution in AML was characterized. Chronic inflammation suppressed TP53 WT HSCs while enhancing the fitness advantage of TP53-mutant cells and promoting genetic evolution.	First noted the importance of chronic inflammation in TP53-mutant AML progression. Facilitated the risk-stratification, early detection and treatment strategies for TP53-mutant leukemia.	[52]
CLL	scRNA-seq	Putative driver SF3B1 mutation was found to dysregulate multiple cellular pathways including DNA damage response, telomere maintenance, and Notch signaling (mediated by KLF8 upregulation, increased TERC and TERT expression, or altered splicing of DVL2 transcript, respectively).	Characterized how SF3B1 mutation functions in CLL progression and offers selective advantages. These pathways can be therapeutically targeted in SF3B1 mutated CLL patients.	[54]
AML	scRNA-seq	Myc targets are upregulated along the progression of AML, among which are splicing factors. The tipping point of HSC transformation into leukemia cells was characterized by dramatically increased splicing factors and unusual RNA velocity. Exon 4 skipping of Tmem134 in high-risk subset resulted in the production of cell-cycle-promoting Tmem134β.	Characterized that Myc-driven CLL progression was related to the RNA splicing events, promoting that the splicing factor may underly important therapeutic targets.	[55]
AML	CITE-seq; ATAC-seq	Flt3-ITD mutation, when cooperates with NUP98 and Runx1 mutations, activates distinct transcriptional programs. Flt3-ITD/Runx1del caused aberrant expansion of myeloid progenitors, while Flt3ITD/NHD13 selectively controlled IFN-I signaling to drive the clonal expansion of the pre-AML population.	Provided insight into how to context-specifically treat pediatric and adult AML, since Flt3-ITD/NHD13 and Flt3-ITD/RUNX1del respectively represent the most prevailing mutation in pediatric and adult AML.	[34]

### Revealing dynamic leukemia phylogeny characteristics

Back in 2015, a study using a self-established single-cell genotyping method revealed that the underlying clonal structure and evolutionary trajectory in AML may be more complex than the bulk data suggested [40]. The power of single-cell sequencing technology in clonal evolution study was strongly depicted in 2019 when Chen et al. suggested the non-linear and parallel clonal evolutionary model of pre-myelodysplastic syndromes (pre-MDS) stem cells to MDS blast or to AML by targeted sequencing [41] (Fig. 3a). It revealed that AML progression was dominated by small stem cell subpopulations that are undetectable in MDS blast but expand dramatically in size during disease progression [41]. This is in parallel with the previously described linear model that MDS is often considered a pre-leukemia state and was transformed to AML by serial mutation gain [42].

In 2020, two studies more comprehensively revealed the clonal evolution trajectories in leukemia both by applying scDNA-seq with cell surface protein immunotyping [15, 43]. By analyzing 146 samples from 123 patients with clonal hematopoiesis (CH), myeloproliferative neoplasms (MPN), or AML, Miles et al. showed the clonal architecture and the evolutionary pattern in myeloid malignancies [15]. It has revealed that AML is primarily controlled by a small number of clones, which often contain co-occurring mutations in epigenetic regulators. Additionally, the clonal size, diversity, and evolutionary trajectory exhibit a growing complexity as the disease progresses from CH or MPN to AML, displaying the traits of co-mutation and differential clonal dominance [15]. Another study by Morita et al. focused specifically on the clonal evolution in AML [43]. It identified the co-occurrence and mutual exclusivity among driver genes. Mutual exclusivity of function-redundant mutations is often observed in different subclones of AML. Using single-cell data, the authors reconstructed the phylogenetic trees, which found that about half of AML patients showed typical linear clonal patterns whereas another half showed a branched pattern with evolutionary convergence [43] (Fig. 3b). The convergent pattern of evolution is not rare in leukemia as another study in CLL also identified the existence of cells with analogous phenotypes despite substantial genetic heterogeneity [44]. Collectively, these studies highlighted the ability of single-cell sequencing to reconstruct the clonal architecture and evolutionary phylogeny, distinguishing the evolutionary model of linear or branched, convergent or divergent patterns in leukemia.

### Revealing the sequential mutational gain of leukemia

Genetic mutation provides cells with the potential to be positively selected and may ultimately lead to clonal dominance [39]. Malignant development may be induced if cells acquire substantial pro-survival mutations that confer unlimited growth and expansion [45]. Thus, understanding the acquisition and accumulation of mutations has been vital in depicting the phylogeny of leukemia. Emerging single-cell studies have enabled us to gain greater insights into the sequential acquisition of mutations, leading to better detection of disease progression and prognosis.

The study performed by Gawad et al. provided a remarkable insight into the initiation and development of ALL [46]. With targeted scDNA-seq, they discovered that large deletions and most structural variants typically occur early in ALL development, followed by single nucleotide variants (SNV) acquisition. De Bie et al. combined scDNA-seq and scRNA-seq to investigate the order of mutation acquisition in T-ALL [47]. Their findings suggested that mutations in certain genes with ambiguous significance may occur early, laying the foundation for later mutation gain. This is followed by intermediate events such as inactivation of CDKN2A/B, T-cell receptor (TCR) gene deletions, and gene fusions. Interestingly, they discovered that mutation in NOTCH1 was a relatively late event in T-ALL [47]. This was in line with another later study using targeted scDNA-seq, implying NOTCH1 mutations were usually acquired at the later stage of T-ALL [48]. However, high heterogeneity of NOTCH1 mutations was also found at diagnosis in their study. The sequential mutation events in ALL were further explained by combining scDNA-seq and protein analysis, where a study found that the lineage-related mutations (ETV6, IKZF1, and PAX5) occurred early, and kinase-activating mutations (JAK1, JAK2, KRAS, NRAS, FLT3) were acquired in a later evolutionary trajectory [49] (Fig. 3c). Similarly, in AML, a study revealed that the order of mutation gain implied epigenetic modifiers such as DTAI (DNMT3A, TET2, ASXL1, and/or IDH1 or IDH2) are the most prevalent AML initiating mutation, and combinations of those mutations (e.g., DNMT3A-IDH2) may contribute to clonal dominance [15] (Fig. 3c). Comparably, mutations in signaling genes such as FLT3, JAK2, and NRAS are often subclonal. When serving as the initiating mutations, these signaling gene mutations may not easily result in large clonal trajectories.

### Characterizing the driver events underlying leukemia initiation and progression

Leukemogenesis of AML may involve stages from CH, MDS, and MPN [15]. The presence of driver mutations was presumed to fuel the transformation from CH to MDS, MPN and finally, AML. MPN is derived from hematopoietic stem cells (HSC) by driver mutation in JAK/STAT signaling genes and exhibits a propensity for transformation to secondary AML (sAML) by additional mutation gain (e.g. TP53, ASXL1, EZH2, SRSF2, IDH1). However, inhibition of JAK2 showed limited effect and didn’t prevent the disease progression [50]. In light of this, one recent single-cell study confirmed DUSP6, a MAPK pathway member, as the driver of leukemic progression and JAK2 inhibition resistance [51]. By scRNA-seq on serial MPN and sAML patients, increased DUSP6 expression along disease progression from MPN to sAML was found, which mediated JAK2 resistance by activating RSK1 and then S6 phosphorylation. Another study shed light on the TP53-mutant MPN by first proposing the role of chronic inflammation as a driver of TP53-mutant leukemic evolution [52]. Utilizing TARGET-seq [53], a single-cell multiomics technology that allows allelic-resolution genotyping, whole transcriptome, and immunophenotypic analysis from the same cell, the authors found that the presence of chronic inflammation induced with both poly(I:C) and LPS promoted the fitness advantage of TP-53 mutant cells, conferring their genomic instability and leading to clonal dominance.

Studies are also highlighting the role of RNA splicing in driving leukemia initiation and progression. A study employing scRNA-seq in CLL has substantiated that the mutated putative driver SF3B1 results in substantial splicing alterations. Consequently, this leads to the dysregulation of DNA damage response and Notch signaling pathways, ultimately conferring apoptotic resistance and selective proliferation to the leukemia cells [54]. The importance of RNA splicing in leukemogenesis was also noted in a recent single-cell study in AML. With a series of longitudinal scRNA-seq data in a Myc-driven AML mouse model, the authors found progressively deteriorated RNA splicing during AML progression, where increasingly higher expression of splicing factors and stronger enriched spliceosome pathway was observed [55]. Notably, an unusually high RNA splicing factor activity was observed at the tipping point of transformation from HSCs to preleukemic and leukemic cells [55].

With the maturity of single-cell sequencing, increasing studies are discovering novel drivers and characterizing the heterogeneity within the driver events in leukemia. For example, by utilizing targeted scRNA-seq and scDNA-seq to reconstruct the phylogeny and subclonal structures in CLL patients, Wang et al. discovered mutated LCP1 and WNK1 as novel CLL drivers, supported by implicating their impact on CLL pathways [44]. Also, a study in AML using the combination of CITE-seq and epigenomic profiling found that Flt3^ITD^ mutation, frequently discovered in all age groups, when combined with NHD13 and RUNX1 mutations, drove distinct transcriptional programs in mice AML model [34]. Flt3^ITD^/Runx1^del^ caused aberrant expansion of myeloid progenitors, while Flt3^ITD^/NHD13 selectively controlled IFN-I signaling to drive the clonal expansion of the pre-AML population. This is of important clinical relevance as Flt3^ITD^/NHD13 and Flt3^ITD^/RUNX1^del^ respectively represent the most prevailing mutation in pediatric and adult AML, providing insight into why pediatric and adult initiating mutations were differentially skewed [34].

Collectively, these findings decipher the underlying events that drove the initiation and progression of leukemia and depicted the mutations that conferred differential clonal dominance in the evolution of leukemia.

## Single-cell analysis provides rigorous high-resolution baselines for defining leukemia heterogeneity

**Fig. 4:**
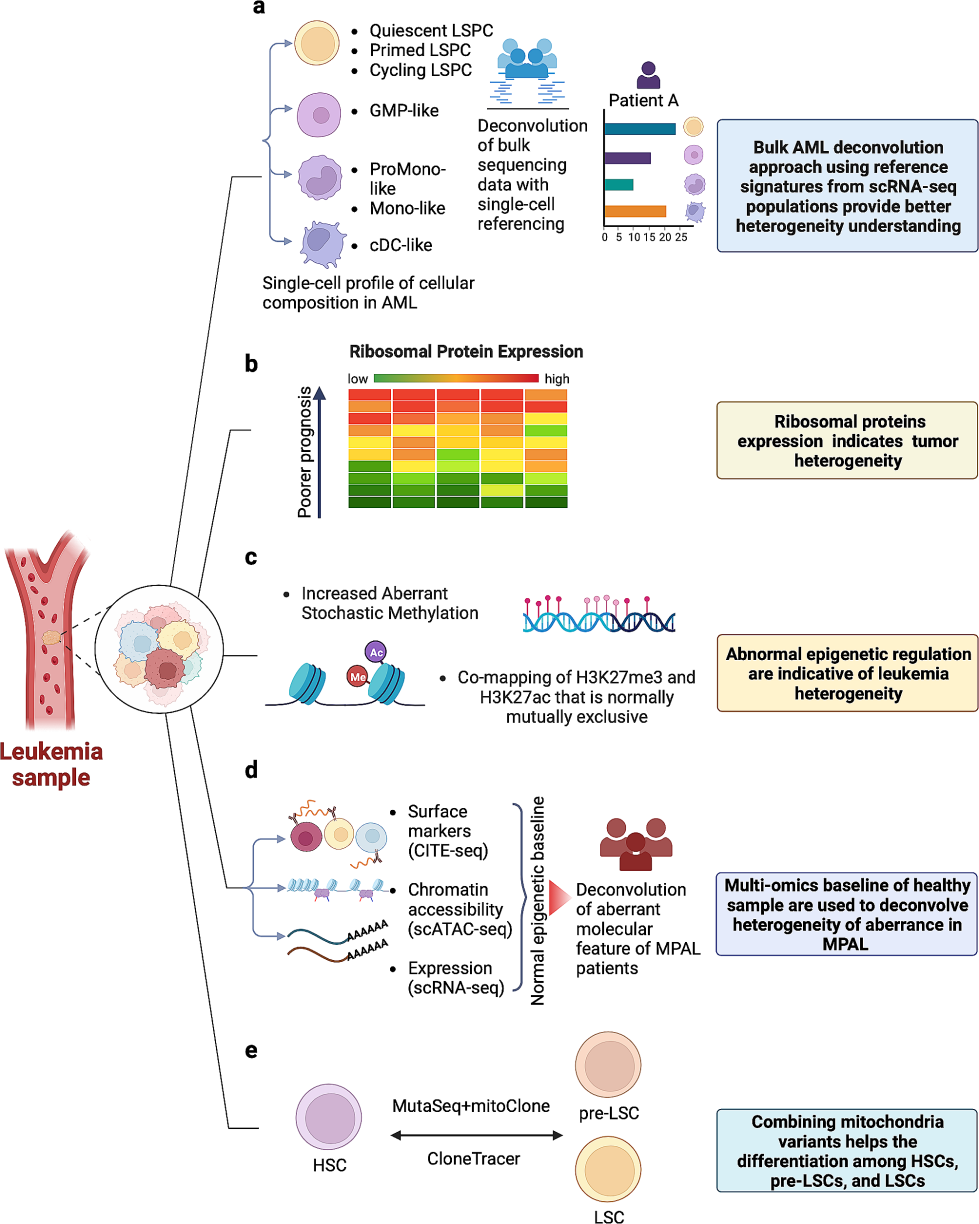
Interrogation on different cellular layers further classifies and defines leukemia in single-cell studies. (**a**) AML heterogeneity was better defined with deconvolution of bulk data by single-cell referencing. By deconvolution > 1000 AML patients bulk RNA-seq data using single-cell referencing, AML composition was converged into four overall classes, Primitive (LSPC-enriched), Mature (Mono-like and cDC-like blasts), GMP and Intermediate (balanced dstribution) and used as references for patient sample [59]. (**b**) Ribosomal protein expression levels are indicative of heterogeneity in prognosis in different leukemia subtypes. Higher expression of RPs may associated with poor outcomes [60, 61]. (**c**) Different malignant epigenetic layers indicate leukemia-specific modifications and provide references for subtyping [67, 68]. (**d**) Multi-omics framework (CITE-seq, scATAC-seq and scRNA-seq) defined the normal epigenetic baseline of healthy blood development and were used to deconvolve aberrant molecular features of MPAL patients [35]. (**e**) Multiple frameworks combining the mitochondrial mutational landscape with transcriptome and genetic mutation information (MutaSeq + mitoClone; CloneTracer) more confidently differentiated HSCs from LSCs [75]

Leukemia is a highly heterogeneous disease with extensive differential subpopulations, which has led to limited effectiveness in targeted therapy and the prediction of prognosis. Conventional classification of leukemia relied on morphological, immunologic, and clinical manifestations, which simply referred to the status of white blood cells in patients [1, 56, 57]. Prior to the advent of single-cell sequencing, the heterogeneity of leukemia cells was primarily identified through flow cytometry. However, this method was hindered by the quality of antibodies and the limited quantity of antibody labels. The emergence of single-cell sequencing technology has expanded our understanding of leukemia cell heterogeneity to an unprecedented level. Specifically, single-cell sequencing technologies have facilitated the development of systemic frameworks that integrate multiomics and inductive algorithms, leading to improved insights into leukemia heterogeneity. These multi-dimensional approaches offer increased accuracy in defining subtle yet important subpopulations that may contribute to drug resistance and relapse and were summarized in (Fig. 4; Table 2).

**Table 2:** Summary of key findings related to heterogeneity indicators or classifying frameworks in leukemia by single-cell sequencing

Leukemia Type	Major Methods	Key Findings	Clinical Relevance	References	
AML	scRNA-seq; Targeted DNA sequencing; Single-cell short/long read sequencing	Machine learning was performed on high-throughput single-cell data and identified six malignant AML cell types, HSC-like, progenitor-like, GMP-like, promonocyte-like, monocyte-like, or cDC-like malignant cells, along the HSC to myeloid axis.	Related the AML developmental hierarchies to genotypes, providing information on how primitive AML cell types are prognosis informative.	[58]	
AML	Bulk transcriptome deconvolution using single-cell references	AML hierarchy was subtyped into four overall classes, spanning Primitive, Mature, GMP, and Intermediate. LSPC cells were divided into Quiescent, Primed, and Cycling LSPC.	Noted that Primitive vs. GMP axes are chemotherapy responsive whereas Primitive vs. Mature axes is associated with drug sensitivity.	[59]	
AML	scRNA-seq; SMRT-seq	AML progenitor cells cluster with novel AML markers associated with dysregulated RP expression were identified.	Highlighted that the high ribosomal protein involved in the p53 pathway in the progenitor cells subtype was associated with poor outcome.	[60]	
cALL	scRNA-seq	Ribosomal protein expression profile is distinctive and inversely correlated with the presumptive ALL developmental state.	Highlighted that ribosomal protein may be considered as a marker for intra-individual heterogeneity in cALL patients.	[61]	
AML	scRNA-seq	C1Q + macrophage-like leukemia subset was identified and verified in multiple patients with AML. C1Q + leukemia cells represent a highly tissue-infiltrative leukemia population and could reconstitute extramedullary infiltration phenotype of AML. C1Q interacts with C1Q–globular C1Q receptor on fibroblasts, regulating the cancer infiltration pathways and promoting the chemoresistance of C1Q + leukemia cells.	Put forwarded that C1Q can serve as a marker for AML with adverse prognosis and the cancer infiltration pathways. Also, C1Q is a great therapeutic target.	[62]	
AML	scRNA-seq	NFIC protein is significantly overexpressed in 69% of acute myeloid leukemia patients, and increased expression of growth and survival genes in monocytes. NFIC knockdown in an ex vivo mouse a pre-leukemic stem cell model decreased their growth and colony formation and increased expression of myeloid differentiation markers Gr1 and Mac1.	Noted that NFIC is an important transcription factor in myeloid differentiation as well as AML cell survival, and is a potential marker for therapeutic targeting in AML.	[63]	
CLL	scRNA-seq; Whole-genome bisulfite sequencing	High level of methylation heterogeneity in CLL arose from stochastic methylation dysregulation.	Identified that dysregulation of methylation is associated with poor prognostic outcome in CLL patients.	[67]	
CLL	Multiplexed single-cell reduced representation bisulfite sequencing (MscRRBS); scRNA-seq; ChIP-seq	Coordination between different layers of CLL epigenome layers and epigenomic expression was disrupted, attributing to cell-cell heterogeneity.	Noted that corrupted epigenetic layers residing in CLL may stochastically activate heterogenous expression programs, associating poor prognosis.	[68]	
Computational framwork	Developed sc-compReg for comparative analysis between disease and healthy samples based on scRNA-seq data and scATAC-seq data	Sc-compReg in CLL samples identifies TOX2 as a key regulator of tumor-specific subtypes.	Enabled the integrative comparison between healthy and disease states based on transcriptomic and chromatin accessibility. Further application in other leukemia subtypes could review more distinct subtypes.	[69]	
MPAL, AML	CITE-seq; scRNA-seq; scATAC-seq	Single-cell epigenetics baseline for healthy blood samples was established, which was used to deconvolve aberrant molecular features of MPAL. 91,601 putative peak-to-gene linkages and transcription factors regulating leukemia-specific genes were identified.	Demonstrated that single-cell multiomics study may provide novel shared molecular mechanisms among different leukemia types for clinical targeting.	[35]	
Experimental and Computational framework	Developed MutaSeq and mitoClone for single-cell targeted mutation analysis of nuclear and mitochondrial genes on scRNA-seq data.	Application of Mutaseq and MitoClone in AML implied that LSC, HSC and pre-LSC can be more confidently distinguished based on the combination of transcriptome, genetic and mitochondrial variants. Genetic mutations can distinguish between healthy and diseased states, and expression profiles can identify stem or progenitor cell states.	Demonstrated that mitochondrial mutations may also indicate leukemia heterogeneity and underlie therapeutic targets.	[75]	
Experimental and Computational framework	Developed Optimized 10X and CloneTracer for clone tracing specifying nuclear and mitochondrial mutation on scRNA-seq data.	Application of CloneTracer to 19 AML patient samples revealed healthy or preleukemic state in a dormant HSC subset. Discovered that LSCs resemble HSCs expression but formed differential-blocked aberrant myeloid progenitors in downstream.	Demonstrated that mitochondrial mutations may also indicate leukemia heterogeneity and underly therapeutic targets. LSCs may be distinguished from HSCs by forming aberrant myeloid progenitors in downstream.	[76]	

### Single-cell transcriptome in defining leukemia subtypes

Leukemia cells exhibit significant heterogeneity, encompassing both primitive, often referred to as leukemia stem cell (LSC), and differentiated cell types. The utilization of scRNA-seq presents an opportunity to precisely partition leukemia cell subgroups, and even further subdivide the LSC cell population, based on transcriptional variations at the single-cell level.

van Galen et al. employed scRNA-seq to establish a hierarchical framework of normal hematopoietic cells using bone marrow cells from healthy volunteers [58]. They utilized this reference to classify AML cells into distinct subgroups, such as HSC-like, progenitor-like, granulocyte-macrophage progenitor (GMP)-like, promonocyte-like, monocyte-like, or conventional dendritic cell (cDC)-like malignant cells, along the HSC to the myeloid axis. Zeng et al. employed the method of self-assembling manifolds to analyze the scRNA-seq data of AML patients from van Galen et al. [59]. They further subdivided leukemia stem and progenitor cells (LSPCs) into Quiescent, Primed, and Cycling LSPCs (Fig. 4a). Utilizing the AML hierarchy established by the scRNA-seq data, they characterized the cellular heterogeneity of more than 1000 AML patients by deconvolution of bulk transcriptome data. Variations between different hierarchies provide different predictive biomarker information, with the Primitive versus GMP axis showing strong prognostic value in terms of chemotherapy outcome, and the Primitive versus Mature axis capturing ex vivo drug sensitivity [59]. This study incorporated the stem-cell feature with the AML hierarchies, providing a novel framework for understanding biomarkers underlying different hierarchical compositions and guiding precise therapeutic selection.

Heterogeneity in the ribosomal proteins (RP) expression of leukemia is also characterized by scRNA-seq and provides prognostic information. In one study that combined scRNA-seq with single-cell single-molecule real-time sequencing (SMRT-seq), it was shown that an AML progenitor cell cluster is associated with dysregulation of RP, characterized by the expression of high-level RP genes and exhibiting poor remission [60]. Another study using scRNA-seq on childhood ALL (cALL) also indicated that the RP expression profile is distinctive and inversely correlated with the transcriptomic heterogeneity in ALL. This could be a common contributor to intra-individual heterogeneity in cALL patients [61] (Fig. 4b). These findings suggest that there may be heterogeneity in the RP expression of leukemia patients and high RP expression indicates poor prognosis. Recently, the advent of scRibo-seq has opened up exciting new possibilities for gaining a deeper understanding of the role of translational heterogeneity in characterizing different tumor subtypes [30].

In addition to RPs, recent scRNA-seq studies are identifying an increasing number of markers that provide insights into heterogeneity. For example, C1Q labeled out the C1Q^+^ macrophage-like leukemia subset, showing tissue-infiltrative ability and could reconstitute the extramedullary infiltration phenotype of AML [62]. The authors showed that C1Q regulates the cancer infiltration pathways and promotes the chemoresistance of C1Q^+^ leukemia cells, which is an adverse prognosis indicator. Another study highlighted the transcription factor NFIC as a promoter of survival and a potential therapeutic target in AML [63]. By scRNA-seq, they demonstrated that overexpression of NFIC in monocytes increased growth and survival gene expression. The ex vivo NFIC knowdown resulted in impaired cell growth and colony formation ability in the MLL::AF9 preleukemic stem cell model.

Collaboratively, these studies decipher the power of using scRNA-seq in deciphering markers for leukemia subsets, thus being therapeutic insightful and prognosis informative.

### Frameworks integrating diverse epigenome layers provides better reference for leukemia heterogeneity

Epigenetic modification plays a crucial role in shaping leukemia heterogeneity [64, 65], where the highly variable epigenetic allele burden has been linked to inferior outcomes in AML [66], and frequent dysregulation of DNA methylation has been observed in CLL [67]. Recently, more studies have incorporated single-cell epigenomics and integrated high-throughput multi-omics data to gain higher resolution and better decode leukemia heterogeneity. For instance, a study revealed that locally aberrant DNA methylation is a stochastic process that becomes more pronounced during CLL progression [67] (Fig. 4c). Another study integrated single-cell DNA methylation sequencing and scRNA-seq with ChIP-seq, establishing a connection between epigenomic modifications and transcriptional profiles [68]. They found the co-mapping of mutually exclusive activating (H3K27ac) and repressing (H3K27me3) histone modifications was more pronounced in CLL compared with normal B cells (Fig. 4c). Most of the co-mapped regions were originally repressed in normal B cells, suggesting an acquisition of activation induced by heightened H3K27ac modification in CLL samples [68].

With the advent of scATAC-seq technology, numerous software tools and pipelines have been developed to integrate scATAC-seq with other single-cell omics techniques, thereby enriching our understanding of chromatin accessibility heterogeneity [69, 35]. One such tool, sc-compReg [69], was developed to integrate scATAC-seq data and scRNA-seq data and was used to build regulatory networks among cell subsets in CLL. They found a tumor-specific B cell subpopulation in CLL that is regulated by the TOX2 gene. Another comprehensive framework combining CITE-seq, scATAC-seq and scRNA-seq was used to deconvolve aberrant molecular features in mixed-phenotype acute leukemia (MPAL) (Fig. 4d) [35]. They discovered 91,601 putative peak-to-gene linkages, as well as transcription factors that govern genes specific to leukemia. For instance, regulatory elements closely linked to the marker gene CD69 were found to be associated with RUNX1 [35].

These studies demonstrate how the integration of single-cell epigenomics sequencing with other single-cell omics methods offers valuable insights into the epigenomic characteristics of various types of leukemia, facilitating a deeper understanding of the disease.

### Combining mitochondria variants confidently separates LSCs from HSCs

Cancer stem cells represent distinct cellular subsets within the heterogeneous tumor, exhibiting striking capacity for initiating disease and underpinning resistance and relapse [70, 71]. In leukemia, LSCs are also thought to have high proliferative potential, are capable of fueling constant tumor growth, and account for sustaining the disease and relapse [71–74]. The journey from normal HSCs to LSCs involves the sequential accumulation of mutations, resulting in the emergence of pre-LSCs and subsequently, LSCs [45]. LSC has been recognized as an unfavorable prognostic indicator. However, effectively targeting LSCs while preserving HSCs has proven to be a formidable task due to their limited abundance and molecular resemblance to HSCs. To overcome this obstacle, a single-cell study adopted a lineage-tracking approach, incorporating both MutaSeq (a scRNA-seq workflow that amplifies nuclear mutations from cDNA) and mitoClone (a clone discovering computational tool using mitochondrial marker mutations) [75], to characterize simultaneous nuclear and mitochondrial mutations within scRNA-seq data, and thoroughly delineate the characteristics of AML LSCs [75] (Fig. 4e). By analyzing the transcriptomes, genetic alterations, and mitochondrial variants, HSCs, pre-LSCs, and LSCs could be discerned, with genetic mutations distinguishing between healthy and diseased states, and expression profiles identifying stem or progenitor cell states [75].

Most recently, one study introduced another set of approaches, the “Optimized 10x” (a scRNA-seq method specifically covering surface antigen expression, nuclear SNVs, and mitochondrial SNV) and CloneTracer (a Bayesian model for clone inferring focusing on nuclear and mitochondria mutational info) for better characterization of LSC and HSC [76] (Fig. 4e). Applying these methods to AML patients confidently distinguished healthy and leukemic cells in 14/19 patients, where mitochondria mutation information is highly informative. By combining data across patients, the authors differentiated healthy and preleukemic cells in a dormant HSC subpopulation [76].

It should be noted that using mitochondrial mutation calling to profile the mutation landscape is less biased since mitochondrial genes are usually consistently highly expressed [75]. The approaches above combining mitochondria variants circumvent the issue of false negatives observed in sole nuclear gene profiling, where cells with low gene expression often exhibit high dropout rates, rendering mutation detection more challenging [75]. The development of these innovative genomic and mitochondrial mutation tracking frameworks not only provides a new dimension for subtyping leukemia but also offers insights into the significance of mitochondrial and genetic mutations in LSC identification.

## Single-cell analysis helps further define the primary tumor microenvironment in natural leukemia development

Despite the intracellular genetic alteration and dysfunction in leukemia accounting for the clonal evolution and expansion is well-established and provided us substantial insights into the development of the disease, emerging studies have noted that the interaction and coevolution of leukemia cells and the cells from the tumor microenvironment (TME), contributes largely to the disease progression [7, 77]. In AML, attempts to link relapse with genetic mutations have shown limited effectiveness, resulting only in extended survival rather than tumor eradication. This underscores the need for deeper insights into non-genetic drivers, which may be situated within the TME [58].

**Fig. 5:**
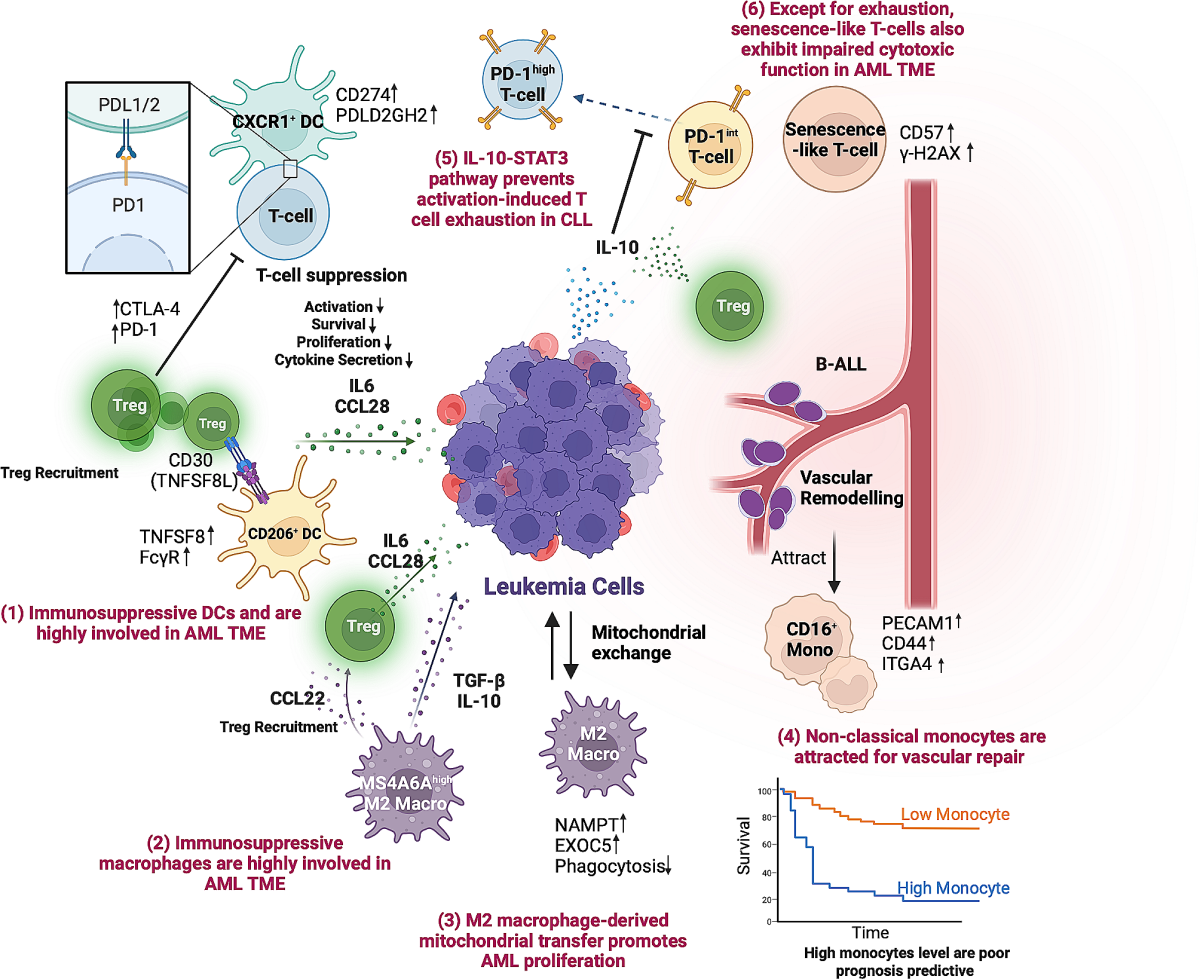
Summary of single-cell studies in leukemic TME. (**1**) Immunosuppressive CD206^+^ and CX3CR1^+^ DC were found in AML TME. CD206^+^ DC recruits Treg by TNFSF8 upregulation and CX3CR1^+^ DC suppresses T-cells by increasing ligands of PDCD1 mediating T-cell suppression (CD274, PDLD2GH2) [81]. (**2**) Suppressive MS4A6A^high^ M2 macrophages were enriched in AML TME and showed Treg recruiting and suppressive signaling function [81]. (**3**) M2 macrophages increased fatty acid oxidation and NAD^+^ generation (NAMPT, EXOC5) and decreased phagocytosis ability in AML TME [83]. Exposure of AML blasts to M2 macrophage resulted in increased mitochondrial metabolism and survival, which is in part due to mitochondrial exchange [83]. (**4**) Non-classical (CD16^+^) monocytes were recruited for repairing the vascular remodeling effect of B-ALL. High-level of CD16^+^ monocytes are poor prognosis predictive [36]. (**5**) IL-10 mediated the PD-1^int^ and PD-1^high^ subtypes of T-cell, preventing the excessive activation and exhaustion of T-cells in CLL TME [91]. (**6**) Senescence-like T-cells (marked by CD57 and γ-H2AX) were found in AML TME, correlating with impaired T-cell cytotoxic effect and poor clinical outcomes [87]

The TME of leukemia is complicated, including mesenchymal stem cells, osteoblasts, endothelial cells as well as immune cells [7, 13, 77]. Multiple recent studies used single-cell studies have delved into investigating the non-immune compartment of the bone marrow niche [78–80]. For example, Baryawno et al. defined the comprehensive single-cell landscape of mice bone marrow in healthy and AML state and found the leukemia cells hindered the process of mesenchymal osteogenic differentiation and decreased the levels of essential regulatory molecules required for normal hematopoiesis [78]. Owing to the limited space capacity, the following section mainly focuses on how the immune compartment of the primary TME (without drug intervention) of leukemia is deciphered by single-cell sequencing (Fig. 5; Table 3).

**Table 3:** Summary of key findings related to single-cell works revealing primary tumor microenvironment in leukemia

Leukemia Type	Major Methods	Key Findings	Clinical Relevance	References
AML	scRNA-seq	The diversity of immunosuppressive CD206 + and CX3CR1 + dendritic cells and different M2 macrophages was defined. Several unique subtypes of TH17-like intermediate population, cytotoxic CD4 + T subset and CD8 + memory-like subset were also identified in AML TME.	Offered a comprehensive AML TME profiling, revealing potential immunotherapy targets.	[81]
AML	scRNA-seq	M2-type macrophage with enhanced oxidative activity and impaired phagocytosis ability in the AML microenvironment. Also, in vitro exposure of leukemic blast to M2 macrophage resulted in the accumulation of CALR-low blast enrichment and the exchange of mitochondria with M2 macrophage. The mechanisms enhanced the survival of AML cells.	Proposed the importance of the interaction of M2 macrophages with AML cells. Revealed potential therapeutic target in terms of metabolism (e.g. FAO/mitochondrial ETC).	[83]
B-ALL	CITE-seq; scRNA-seq	Monocyte abundance is poor prognostic predictive in B-ALL. Non-classical (CD16+) monocyte was attracted by B-ALL and Anti-CSF1R therapy targeting CD16 + monocytes improved the therapeutic outcomes.	Noted that the non-classical monocyte predicts patient survival. Targeting CSF1R of these monocytes together with TKI improved therapeutic outcomes in animal models, revealing a potential therapy combination.	[36]
B-ALL	scRNA-seq	Changes in AP-1-regulated genes were observed in normal pro- and pre-B cells at an early stage of B-ALL. GMP showed tumor suppressor Neat1 downregulation. Monocyte-dendritic precursors (MDP) were continuously active during disease progression. Monocytes increased the interaction with GMP and MDP during progression.	Noted that targeting the MDP, GMP, and monocytes may improve therapeutic outcomes in B-ALL.	[85]
CLL	scRNA-seq	The difference in the number of exhausted CD8 + T cells was significantly larger between the healthy donors (HD) and MBL than between MBL and CLL. Early intervention of ibrutinib can largely reverse the immune dysfunction.	Demonstrated the need for early intervention of CLL by immunotherapy.	[86]
AML	scRNA-seq	Senescent-like CD8 + T-cells were impaired in dealing with AML blasts. Defined a new set of immune effectors dysfunction (IED) signatures that are associated with the adverse outcome and immunocheckpoint unresponsive TME.	Revealed that senescent-like T cells may also be an underlying treatment target. IED scores helped the AML-risk stratification and facilitated the identification of personal treatment targets.	[87]
B-ALL, AML	scDNA-seq	T-cells acquired the exhaustion/dysfunction signature by chronic immune activation in pediatric leukemia TME, manifesting as the attrition of naïve T cells and TCF1 + stem-like memory T cells, and the terminal differentiation of effector T cells. NK cells also expressed a signature of exhaustion, especially in AML.	Noted that although pediatric leukemia has a shorter natural history of tumor exposure, immune cell exhaustion/dysfunction is still a common event and is negatively correlated to the clinical outcomes.	[90]
CLL	scRNA-seq; scATAC-seq	PD-1int subset that was still functional and PD-1hi subset that was exhausted was identified in CLL TME. IL-10 signaling moderates the PD-1 expression through IL-10R-STAT3 pathway and sustains anti-tumor immunity by preventing excessive exhaustion.	Proposed that combining IL-10 with checkpoint blockade therapy may improve the clinical outcome in CLL patients.	[91]
CLL	scRNA-seq	BCL-2 expression was significantly increased in the T cells of CLL patients and associated with increased regulatory T-cells, exhausted cytotoxic T lymphocytes (CTL) signature, and increased T-cell adhesion.	Showed that BCL2 expression in T-cells is associated with immunosuppressive TME. BCL2 may be an underlying therapeutic target.	[92]
CLL	scRNA-seq	CLL progression mainly occurs in the lymphatic nodes (LN) and is associated with suppressive T-cell states. A small population of activated CLL cells progressed in the lymph nodes. Poor outcome was associated with activated CD4 + memory T cells and M2 macrophages in LN. T-cell inflamed microenvironment was progression inhibitive for the tumor.	Attributed the shorter time-to-first-treatment in CLL patients to increased proportion of activated CLL cells. These cells are potentially more effective in recruiting a tumor-supportive TME, thereby accelerating disease progression.	[95]

### Revealing the profile of myeloid cells residing leukemic TME

The malignant leukemia cells in the bone marrow (BM) profoundly remodel the microenvironment and interact profoundly with immune cells. The frequent appearance of myeloid immune cells in leukemia TME, such as dendritic cells, macrophages, and monocytes, have implicated their vital importance [7]. One study comprehensively investigated AML BM using scRNA-seq and highlighted the heterogeneity in myeloid immune cells [81]. This study found the CD206^+^ and CX3CR1^+^ dendritic cell subsets showed an increase in AML patients and may be involved in expanding the Treg population and suppressing the T cell cytotoxic function by producing multiple immunosuppressive cytokines [82]. (Fig. 5). M2-type macrophage subsets marked by MS4A6A^high^ and CD163^high^ were also enriched. MS4A6A^high^ macrophage is a common subset expressing Treg-attracting chemokine CCL22 whereas CD163^high^ was identified as a novel subset [81]. The role of M2 macrophage was also noted in one recent study in AML [83]. By the integration of scRNA-seq, flow cytometry, and immunohistochemistry, the tumor-supportive M2 macrophages with enhanced fatty acid oxidation and NAD^+^ generation were observed, accompanied by impaired phagocytosis ability (Fig. 5). Also, a mechanism of direct mitochondrial transfer between the M2 macrophage and AML blast was observed, which enhanced the mitochondrial metabolism of leukemic blasts and promoted their proliferation [83]. The study revealed the potential targeting strategy in metabolism by targeting fatty acid oxidation or mitochondrial electron transport chain (ETC) in tumor-associated macrophages.

Monocytes, being an integral part of the innate immune system, exhibit noteworthy regulatory effects pertinent to the development of cancer [84]. Recent single-cell studies highlighted the monocyte-related role in promoting tumor survival [36, 85, 86]. One study investigated the immune cells from healthy donors and B-ALL patients with scRNA-seq and CITE-seq and unveiled that B-ALL orchestrated the preferential differentiation of non-classical monocytes (identified as CD14^dim^, CD16^+^) through interactions with vascular endothelial cells, thereby reversing the alterations in vessel diameter induced by B-ALL cells [36] (Fig. 5). The non-classical monocytes were observed to promote B-ALL survival without suppressing T cells and were predictive of unfavorable prognosis in B-ALL patients [36]. The distinctively evident communications of monocytes with tumor cells were also found in B-ALL and CLL [85, 86]. However, it is also worth noting that the specific study on B-ALL highlighted the interaction between monocytes and monocyte precursors [85]. These findings underscore the importance of targeting monocyte-related regulatory pathways, such as CSF1R blockade, as potential therapeutic interventions.

### Revealing the heterogeneity within the T-cell exhaustion state

T cell exhaustion poses a significant challenge to effectively clearing tumors [87]. The upregulation of exhaustion markers such as PD-1, LAG3, CD200, and TIM3 on CD8^+^ T cells signifies the loss of cytokine signaling and cytotoxic dysfunction, indicating an exhausted state [88]. However, checkpoint blockade therapy has not shown desired efficacy in AML and CLL [89]. Understanding the underlying mechanisms behind this requires further molecular insights, which can be provided by recent single-cell studies.

Recently, studies have revealed widespread evidence of dysfunctional T cell states in leukemia, including the accumulation of attrition of naive T cells, activation of Tregs (regulatory T cells), terminally differentiated cytotoxic T cells and exhausted T cells [81, 90–92]. The BCL2 family consists of apoptosis regulators that promote cell survival and have been linked to various forms of malignancy [93]. However, Liu et al. found that heightened BCL2 expression in the T cells of CLL patients was also associated with increased occurrence of CD8^+^ cytotoxic T cells exhibiting exhaustion (PD-1^+^, TIM-3^+^) and a higher proportion of Tregs [92]. Heterogeneity within the exhausted CD8^+^ T cell population of CLL was further revealed by Hanna et al., identifying the PD-1 intermediate expression (PD-1^int^) subset that was still functional and PD-1 high expression (PD-1^hi^) subset that was exhausted [91]. Further investigation revealed that the IL-10R-STAT3 signaling pathway moderates the balance between PD-1^int^ and PD-1^hi^ subsets by maintaining normal chromosomal accessibility landscape and NFAT: AP-1 cooperativity, thereby preventing the excessive activation of CD8^+^ T cells and transformation to a terminal PD-1^hi^ exhaustion state [91] (Fig. 5). These findings suggest that enhancing IL-10 signaling could potentially enhance the efficacy of checkpoint blockade therapy in CLL by preventing the transformation of CD8^+^ T cells into a terminal exhaustion state.

In general, exhaustion of T cells is typically a consequence of prolonged or chronic exposure to persistent antigens [88]. However, scRNA-seq analysis has suggested that the dysfunctional state of T cells may already be present at the precursor phase of CLL, known as monoclonal B-cell lymphocytosis [86], indicating the need for early induction of immunotherapy during CLL progression. Also, pediatric leukemias (AML and B-ALL), which naturally have lower neoantigen load and immunogenicity compared to adult tumors, were observed to have a high degree of T cell and NK cell exhaustion [90]. By interrogating the single-cell mass cytometry and scRNA-seq, the study discovered the depletion of stem-cell-like TCF1^+^ T cells both in pediatric B-ALL and AML [90]. Furthermore, a more pronounced dysfunction of NK cells was observed in AML compared to B-ALL, indicating the necessity for treatment heterogeneity when dealing with these two types of leukemia [90].

### Proposing the roles of senescent-like T-cells in TME

It has been suggested that not only exhausted T-cells but also senescent-like T-cells are associated with poor outcomes in cancer [87]. Exhaustion and senescence share some properties but are functionally independent, marked by differential activated signaling pathways [94]. To address the knowledge gap regarding the contribution of T cell senescence to the anti-immunotherapy effect, Rutella et al. conducted a study combining bulk and single-cell RNA sequencing to characterize how AML cells promote the senescence-like CD8^+^ T cells [87]. They found that AML blast can lead to the expression of typical senescence markers CD57 and γ-H2AX on CD8^+^ T cells, primarily through bystander modulation (Fig. 5). They also defined a new immune effector dysfunction (IED) score, which proved to be powerful in risk stratification and determining prognosis [87]. This study provides insights into strategies for overcoming blockade-unresponsive therapy and improving the prognosis of AML patients.

### Relating immune-associated TME with leukemia progression

Tumor progression is not only influenced by intrinsic alterations but also by extensive interactions between TME and the tumor cells [85]. Integrating bulk and single-cell transcriptome profiling in the paired peripheral blood and lymphatic node, a study identified unidirectional CLL clonal progression from activation to quiescence, which majorly occurs in the lymphatic node and is correlated to immunosuppressive T cell state [95]. The active-state tumor cells represented were positively related to activated CD4^+^ memory T cells and M2 macrophages in LN, which predicts a poor prognosis. Also, the T cells-inflamed immune microenvironment was progression-inhibitive, by suppressing the clonal outgrowth of CLL [95]. Apart from T cells, another study in B-ALL revealed the consistently reduced expression of tumor-suppressing non-coding RNA Neat1 in GMP cells [85], which was shown to induce cancer initiation and drug resistance [96, 97]. These findings suggest that leukemia tumor progression is a dynamic and interactive process involving coevolution with the TME.

## Single-cell studies provide further insights into drug effects and help uncover drug-resistant mechanisms

Leukemia is characterized by dysregulation of cellular pathways and significant intrinsic heterogeneity, posing challenges to effective treatment strategies. Despite the development of drugs like tyrosine-kinase inhibitors (TKIs) have dramatically improved the clinical outcomes of patients, resistance and relapse independent of the BCR-ABL fusion protein are still common issues [98, 99]. Also, the standard treatment regimen for AML (anthracycline + cytarabine, DA) has been applied for more than four decades without a new consensus on how to tackle the disease [100]. Thus, it is urgent to understand leukemia’s molecular features to unravel the mechanisms of drug resistance at a higher resolution by single-cell studies. Here, we emphasized the usage of single-cell sequencing in dissecting three major aspects that were substantially associated with relapse: leukemia stem cells (Table 4), the therapeutic tumor microenvironment (Table 5), and therapy-induced clonal evolution (Table 6).

**Table 4:** Summary of key findings related to drug resistance mechanisms induced by leukemia stem cells using single-cell sequencing

Leukemia Type	Major Methods	Key Findings	Clinical Relevance	References	
CML	scRNA-seq	Consistent and distinct expression of CD93 was observed on a lin − CD34 + CD38 − CD90 + CML LSC population and showed stem cell characteristics and quiescent characters. CD93 + LSCs subpopulation persisted in relapsed CML patients after the withdrawal of TKI treatment.	Showed that the CD93 is selectively and consistently expressed at the CML LSCs subpopulation, which indicates poor TKI responders.	[99]	
CML	scRNA-seq; single-cell targeted mutation analysis in DNA	TGF-β and TNF-α were dysfunctional in both BCR-ABL- LSCs and BCR-ABL + LSCs. Long-term TKI treatment selected a quiescent LSC subpopulation, showing TGF-β, TNF-α, and IL-6–JAK-STAT gene enrichment. RUNX1 mutation in LSC was observed for patients entering blast crisis.	Revealed a series of prognostic markers including RUNX1 and provided indicators for TKI response.	[102]	
CML	scRNA-seq	Poor imatinib responders enriched patient-specific pre-treatment stem/progenitor cells compared with responders. The stem cell feature of LSCs was present at diagnosis rather than acquired by the treatment.	Indicated that the stem cell of LSCs feature was intrinsic rather than acquired during TKI therapy in CML, revealing the need for early intervention for LSCs. .	[103]	
AML	scRNA-seq	Reprogramming of stem/progenitor-like cells into quiescent stem-like cells may provide AML with resistance during chemotherapy. Upregulation of CD52 and LGALS1 marking quiescence was observed, where CD52-SIGLEC10 interaction between QSCs and monocytes underlie the mechanism for immune evasion and resistance. Also, the LGALS1 inhibitor could help eliminate QSCs and enhance the chemotherapy in patient-derived primary AML cells.	Identified the quiescence marker, LGALS1, as a promising target for chemoresistant AML.	[104]	
AML	scRNA-seq	The proliferation and self-renewal LSCs subpopulation was separated in AML, where Cd69 High LSCs were capable of self-renewal and Cd36 High LSCs were highly proliferative.	Noted that simultaneously targeting the self-renewal and proliferation in LSCs is essential for treating AML.	[105]	
AML	scRNA-seq	C-Kit + B220 + Mac-1- and c-Kit + B220 + Mac-1 + LSC subpopulations were found in Setd2-/- AML, where the Mac + subpopulation was resistant to doxorubicin plus cytarabine (DA) treatment with the activation of RAS pathway.	Showed that treatments combining DA and RAS pathway targeting may improve the clinical outcome of AML.	[106]	
AML	scRNA-seq	Induced by chemotherapy, AML cells depleted LSCs and entered a senescent-like phenotype. This kind of senescence was transient with increased engraftment ability. Entering the senescence-like phenotype was dependent on ATR. Post-senescence AML cells increased stem cell potential and conferred relapse.	Proposed that the stem cell feature of AML presented at relapse may be the consequence rather than the reason for relapse. Targeting the senescent-like feature by ATR may underlie therapeutic effectiveness.	[107]	
AML	CITE-seq	A novel phenotype of monocytic LSC (m-LSC) was discovered, distinguished by CD34-, CD4+, CD11b-, CD14-, CD36-, driving relapse/refractory response in venetoclax-based treatment. This m-LSC is developmentally and clinically distinct from the more well-described primitive LSC (p-LSC) but can co-exist in the same AML patient. The authors found unique enrichment purine/pyrimidine metabolism selective sensitivity to cladribine in m-LSCs.	Offered insight into venetoclax-based treatment relapse and indicated that co-targeting p-LSCs and m-LSCs may be clinically important in treating AML.	[37]	

**Table 5:** Summary of key findings related to drug resistance mechanisms induced by tumor microenvironments using single-cell sequencing

Leukemia Type	Major Methods		Key Findings	Clinical Relevance	References
AML	Paired scRNA-seq and TCR repertoire profiling	TCR repertoires of CD8 + T cells expanded in responders or patients with stable disease after PD-1 blockade treatment and contracted in therapy-resistant patients. GZMK expression and stem-cell feature were observed in the T-cells of responders. Chr7/7q loss was a marker for resistance to PD-1 blockade therapy.		Noted the importance of TCR repertoires. TCR repertories were changed during therapy and indicated treatment response. Chr7/7q was identified as a prognostic indicator for PD-1 blockade therapy in AML.	[38]
AML	scRNA-seq, scDNA-seq, bulk TCRβ sequencing	Combined therapy of anti-PD-1 (pembrolizumab) and hypomethylating agent (decitabine) was feasible and had the best response of stable diseases or better in 6 of 10 patients. Clonal expansion of CD8 + effector memory T cells with PD-1 expression was associated with immune-related adverse events.		Proposed that adding pembrolizumab to current decitabine therapy was clinically feasible in patients with relapsed AML.	[114]
CML	scRNA-seq;	Dasatinib induced the terminal differentiation and exhaustion of CD8 + T cells and NK cells, where the addition of IFN-α reversed this process and increased the number of unique putative epitope-specific TCR clusters.		Supported that the combination of IFN-α with TKI therapy will improve the therapeutic outcome.	[115]
AML	scRNA-seq; CITE-seq; ChIP-seq; ATAC-seq	A novel immunoregulatory effect by histone deacetylase inhibition (HDACi) was associated with the IFN-α pathway. Plasmacytoid dendritic cells (pDC) produce IFN-α after HDACi treatment with increased H3K27 acetylation at the IFN gene. Depletion of pDCs impaired the therapeutic efficiency of HDACi.		Noted that the epigenetic activation of pDCs by HDACi enhances antitumor immunity, suggesting further invention of immunotherapies for epigenetic modulation in pDCs.	[116]
T-ALL	scRNA-seq	T-ALL patient–derived tumor xenografts (PDXs) models were developed. Screened out 39 drugs from 433 clinical-stage molecules using the PDXs model. Discovered that endothelial cells (ECs) and T-ALL cells interact reciprocally, mitigating drug responses in T-ALL PDXs.		Ultimately discovered 5 effective drugs from the drug screening and tested in vivo with therapeutic effects. First developed a T-ALL/EC platform that can help elucidate the leukemia-microenvironment interactions with endothelial cells.	[117]

**Table 6:** Summary of key findings related to drug resistance mechanisms induced by clonal expansion of leukemia using single-cell sequencing

Leukemia Type	Major Methods	Key Findings	Clinical Relevance	References
AML	scRNA-seq	RNA-based clonal evolution tracking was conducted on AML LSCs from matched pre- and post-treatment samples. Commonly evolved signaling networks mediating metabolism, apoptosis and chemokine signaling evolved and became the signature of relapsed samples.	Identified that co-targeting BCL2 and CXCR4 signaling may help improve therapeutic response.	[118]
CLL	Targeted scDNA-seq	After BTK and BCL2 targeting agent (TA) treatments, mutual exclusivity of clonal architecture was observed among multiple resistance mutations to the same targeting therapies. Also, the co-occurrence of multiple novel mutations conferred resistance to dual TA treatment.	Proposed that CLL progression after dual TA treatment is complex but consistently oligoclonal. Different clones have distinct identifiable resistance mechanisms.	[119]
CLL	scRNA-seq; scATAC-seq; mtscATAC-seq	MtDNA mutation was stable over the years and largely changed under strong selective pressure such as allo-HSCT or chemotherapy. The Chromatin state of CLL was also changed (SPIB, SPI1 depletion) and higher expression of CXCR4 was observed at relapse.	Marked that mtDNA mutations and chromosomal state as a clonal tracking method for leukemia progression.	[120]
CLL	scRNA-seq; ATAC-seq	Consistent regulatory program in BTKi treatment was observed starting with a sharp decrease of NF-κB binding, continued with decreased activation of lineage-defining transcription factors and the final acquisition of a quiescent signature.	Established the time-dependent expression and gene regulatory response after BTKi treatment, offering a new method for treatment monitoring.	[121]
CLL	Computational system combining scRNA-seq and DNA barcoding	An integrative lineage tracing system was developed (ClonMapper), which combines DNA barcoding scRNA-seq. ClonMapper identified CLL subpopulations with distinct molecular features and survivorship trajectories during chemotherapy.	Associating CXCR4, Wnt and Notch signaling with the higher survival rate of CLL after chemotherapy.	[123]
CLL	scRNA-seq; WES; Methylome sequencing	Pre-existing stem-cell-like subpopulations that conferred resistance after allo-HSCT treatment in early relapse samples. Early relapse featured a stable genome whereas late relapse featured strong genetic evolution, neoantigen depletion, and epigenomic instability.	Described clinical kinetics post-HSCT treatment in CLL.	[125]
ALL	scRNA-seq	Stem cell properties with the quiescent feature, and activation of glucocorticoid response were marked as relapse-initiating subpopulation in MLL-rearranged infant ALL (MLL-r iALL).	Provided insights for the risk stratification of MLL-r iALL	[127]
B-ALL	sc-CyTOF, RNA-seq	Coordination between the glucocorticoid receptor pathway and B-cell developmental pathway was identified. The BCR signaling pathway was enriched during GC treatment, marked by activation of PI3K/mTOR and CREB signaling and accounted for the GC resistance. Dasatinib targets these active signaling and eliminates the GC resistance.	Indicated that the combination of GCs and TKIs may improve therapeutic outcomes in B-ALL patients.	[128]
AML	scDNA-seq	AML Patients treated with VEN-based therapy with higher response rates were associated with NPM1 or IDH2 mutations, and poor responses or relapse were associated with TP53 loss or kinase activation, particularly FLT3 activation.	Provided insights for the risk stratification and prognostic prediction with older AML patients receiving venetoclax-based combination therapies.	[133]
AML	scDNA-seq	VEN + AraC treatment induced adaptive resistance in AML, characterizing changes in oxidative phosphorylation, electron transport chain complex I (ETCI) and the TP53 pathway. ETC inhibition, pyruvate dehydrogenase inhibitors and mitochondrial ClpP protease agonists improved therapeutic outcomes in VEN + AraC-resistant AML samples.	Noted that the mitochondrial and energy-related inhibitors may be clinically combined with VEN-based therapy to improve therapy outcomes.	[134]
AML	scDNA-seq; DNA methylation profilling	RAS/MAPK pathway, which leads to increased MCL-1 protein expression was the major mechanism for resistance to the VEN. MCL-1 protein maintained the respiration in VEN-resistant cells.	Identified the importance of combining VEN and the RAS/MAPK/MCL-1 pathway inhibitor for AML treatment. This strategy may overcome the VEN resistance and improve AML patient survival.	[135]
CLL	CITE-seq; single-cell short and long read RNA sequencing	Multilayered resistant mechanism was observed in VEN-resistant CLL, including mutations in BCL2 and MCL1 amplification. Universal upregulation of the MCL1 gene was observed, driven by NF-κB pathway activation, and this stopped after discontinuation of VEN therapy.	Proposed that the NF-κB pathway targeting may be a key for improving clinical outcomes in VEN-resistant CLL.	[136]

### Uncovering drug effects and resistance by decoding LSCs

**Fig. 6:**
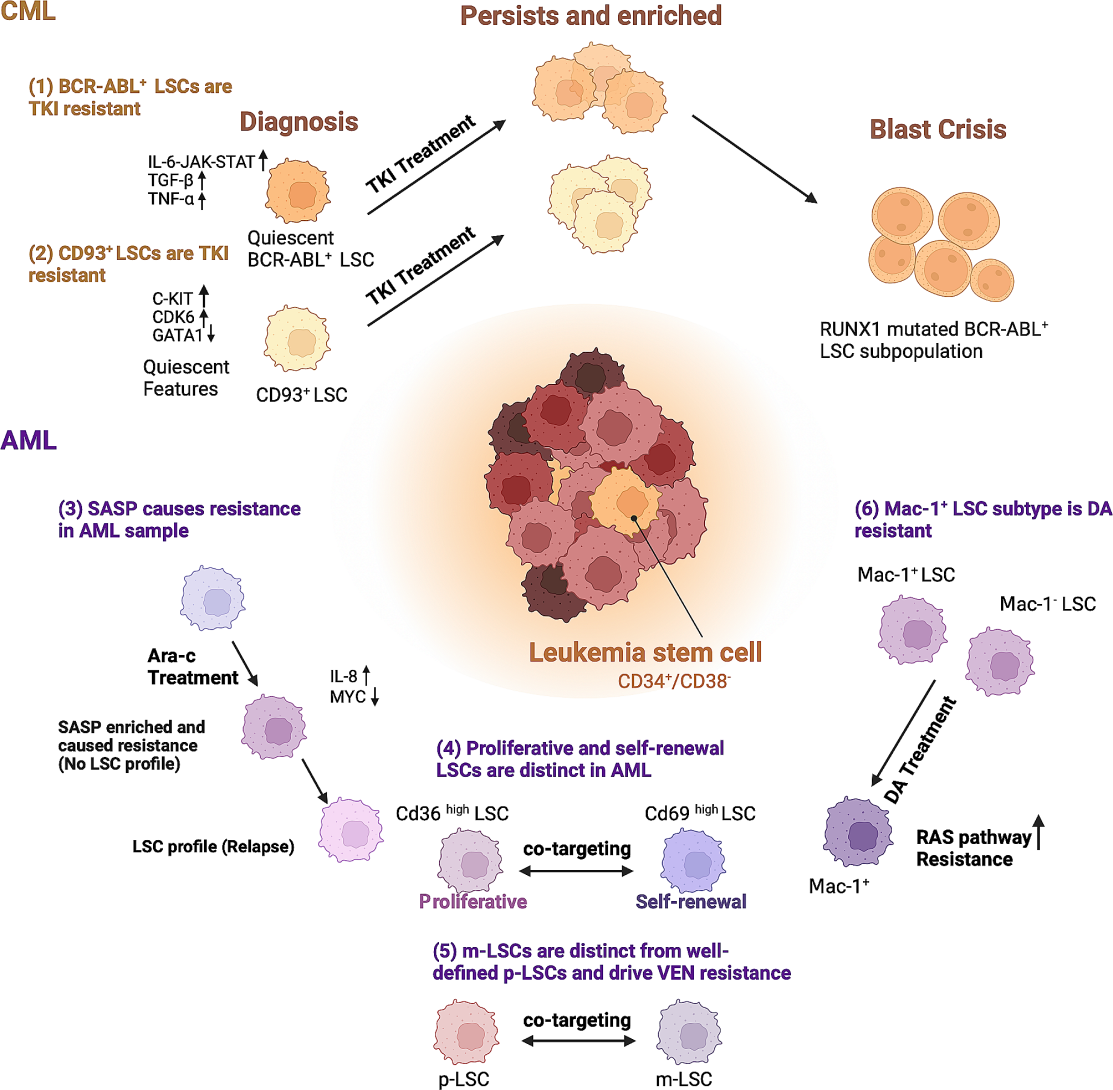
Heterogeneity within LSCs mediated therapy resistance. **(Upper, CML)** (**1**) Quiescent BCR-ABL^+^ LSC subpopulation, with inflammatory-associated gene upregulated at diagnosis, persisted during TKI treatment. RUNX1 mutation gain was linked to the blast crisis in BCR-ABL^+^ LSC [102]. (**2**) CD93 was found to be selectively and persistently expressed in an LSC subpopulation, with quiescent gene upregulated at diagnosis. This LSC subpopulation persisted during TKI [99]. **(Lower, AML)**. (**3**) AML LSC profile at relapse was presumed to be the consequence of recovery from senescence-associated secretory phenotype (SASP), a phenotype induced by Ara-c treatment [107]. (**4**) Proliferative (Cd36^high^) and self-renewal (Cd69^high^) subtypes were found to be distinct in LSCs. Only targeting proliferation or self-renewal pathways caused resistance, and simultaneous targeting improved therapeutic outcomes [105]. (**5**) Monocytic LSC (m-LSC) was distinguished from previously well-defined primitive LSC (p-LSC) and drives resistance to venetoclax(VEN)-based treatment [37]. Co-targeting m-LSCs with cladribine may be clinically important. (**6**) Mac-1 was found to be differentially expressed in LSCs. Mac-1^+^ subtype is DA resistant with higher RAS pathway activation [106]

LSC was conventionally identified by CD34^+^/CD38^−^ surface marker. Multiple heterogeneities have been found within LSCs that account for the disease progression, alterations in the microenvironment, and induction of therapeutic relapse [72, 101]. To understand the mechanisms behind drug resistance caused by LSCs, a series of studies utilizing single-cell sequencing have been conducted, which are summarized in Fig. 6; Table 4.

Early in 2017, Giustacchini et al. utilized scRNA-seq and single-cell-based mutation detection targeting the BCR-ABL gene to disclose the heterogeneity of LSCs in CML [102]. Through single-cell analysis of serial samples from BCR-ABL^+^ LSCs taken from patients undergoing long-term (> 1 year) TKI treatment, a quiescent subpopulation was identified. This subpopulation was already present at diagnosis, persisted and enriched during the treatment, and showed gene enrichment related to TGF-β, TNF-α, IL-6, and JAK-STAT signaling pathways (Fig. 6) [102]. Also, the scRNA-seq data of those patients entering blast crisis (BC) revealed the presence of RUNX1 mutations in distinct BCR-ABL^+^ LSCs subclones. This discovery implied that RUNX1 mutation could serve as a poor prognostic marker [102].

In 2020, Zhang et al. performed scRNA-seq on cells derived from four CML patients treated with TKIs and found poor responders were enriched with pre-treatment stem/progenitor cells compared with responders [103]. Trajectory analysis validated the presence of tumor cells with primitive characteristics prior to treatment, indicating that the resistance to TKIs may be intrinsic rather than acquired through treatment [103]. The work conducted by Kinstrie et al. also supported the notion of intrinsic resistance in LSCs. Their study identified the persistent and selective expression of CD93 within a lin^−^CD34^+^CD38^−^ CD90^+^ CML LSC subpopulation, which showed higher proliferative potential and could persist TKI treament [99] (Fig. 6). These findings underscore the significance of CD93 as a potential prognostic marker for TKI treatment [99]. Most recently, the importance of quiescence in LSCs was marked again in one recent study by Li et al. [104]. Researchers found that reprogramming of proliferating stem/progenitor-like cells into quiescent stem-like cells (QSCs) may confer AML resistance during chemotherapy [104]. By longitudinal scRNA-seq of 6 AML patients during cytarabine (Ara-c)-based treatment, upregulation of CD52 and LGALS1 (the marker for QSC phenotype) was observed, where CD52-SIGLEC10 interaction between QSCs and monocytes may underly the mechanism for immune evasion and resistance [104].

Except for quiescent LSCs, Sachs et al. found the two subsets of AML LSCs in mouse models with respectively high expression of Cd69 and Cd36 (Fig. 6) [105]. The Cd36-high subpopulation showed stronger self-renewal but proliferative disabled whereas the Cd69-high subpopulation had a stronger proliferative capacity but could not initiate leukemia development [105]. This highlighted the clinical relevance of targeting both self-renewal and proliferation as essential therapeutic strategies for AML [105]. Applying scRNA-seq, another study identified two subsets of LSCs featured by c-Kit^+^ B220^+^ Mac-1^−^ and c-Kit^+^ B220^+^ Mac-1^+^, respectively (Fig. 6) [106]. The c-Kit^+^ B220^+^ Mac-1^+^ cells displayed intrinsic resistance when subjected to the DA treatment in vivo, with the higher activation of the RAS pathway. Thus, the combination therapy involving DA and RAS inhibitors effectively impeded disease progression in the murine model [106].

Another innovative discovery by Duy et al. indicates that the relapse of AML is mediated by a resilient subpopulation exhibiting a senescence-like phenotype, regardless of LSC status [107]. The authors found that AML cells were induced to a senescence-associated secretory phenotype (SASP) by ATR signaling in vitro and in vivo after treatment of Ara-c (Fig. 6). Importantly, these senescence-like cells exhibited remarkable engraftment ability and could repopulate AML. (Fig. 6). Surprisingly, there is no enrichment of LSC genes at the nadir but at relapse in senescence-like cells, suggesting that LSC programming may be enhanced in following treatment induction. This implies that the enrichment of stem-cell features at relapse may be a consequence of chemotherapy rather than a cause of chemotherapy tolerance, highlighting ATR inhibition as a potential therapeutic approach [107].

Most recently, with CITE-seq, a study noted the existence of a novel type of monocytic LSC (m-LSC) in AML, driving the refractory/relapse response when treated with venetoclax + azacidine [37]. This kind of m-LSC exhibited a unique immunophenotype (CD34^+^, CD4^+^, CD11b^−^, CD14^−^, CD36^−^) which is fundamentally different from the previously well-described primitive LSC (p-LSC) type [108, 59] while these two types of LSCs can co-reside in the same AML patient (Fig. 6). Gene expression signature implied that the pyrimidine and purine metabolism pathway was enriched in m-LSCs compared to p-LSCs. Importantly, inhibiting purine-based DNA/RNA synthesis by cladribine showed a selectively strong effect in eradicating the m-LSCs, demonstrating a new therapeutic target.

The exact elucidation of how LSCs function in the resistance to therapeutic approach has yet to be determined; nevertheless, the potency of single-cell sequencing in ascertaining the heterogeneity and corroborating specific markers in LSCs has been delineated in these investigations.

### Revealing drug effect and resistance by decoding resistant tumor microenvironment

The TME plays a pivotal role in the initiation, progression, and metastasis of tumors, and has been increasingly identified to be an important therapeutic target in cancer, raising wide clinical interests [109, 110]. Evidence highlighting the importance of TME in leukemia progression has continuously emerged [111], and presented potential therapeutic targets [112]. Recently, serial studies applying single-cell sequencing have helped reveal the drug effects and resistant mechanisms by interrogating TME, especially immune cells (Table 5).

Checkpoint blockade therapy has shown improved outcomes in solid tumors, whose effect, however, was moderate and limited in treating AML [113]. To explore the resistance mechanisms of checkpoint blockade therapy, two studies combined single-cell TCR sequencing, immune profiling, scRNA-seq, and CITE-seq on AML patients treated with the combination of hypomethylating agents and PD-1 inhibitor [38, 114]. One study observed that the CD8^+^ T cell repertoires expanded in patients who responded positively to the drugs or exhibited stable disease whereas the repertoires contracted in resistant patients [38]. Notably, single-cell-based CNV analysis identified Chr7/7q loss as a marker indicating poor response to checkpoint blockade therapy [38]. However, another study found that T cells experienced clonal expansion in patients with immune-related adverse effects (irAEs), but not in those who exhibited antileukemic responses [114]. The expanded clones were mainly composed of CD8^+^ effector memory T cells with highly expressing both PD-1 and cytotoxic-related genes.

Cytokines play an important role in leukemia TME and may underlie therapeutic importance. One study in CML also implied TCR-seq and scRNA-seq to decipher the TME of CML patients undergoing Dasatinib plus IFN-α treatment [115]. This study showed that Dasatinib induced terminal differentiated NK and CD8^+^ T cells. However, the addition of IFN-α reversed this maturation process and restored the immunological function of NK and CD8^+^ T cells. Also, the inclusion of IFN-α broadened the T cell repertoires and enhanced costimulatory interactions with B cells and monocytes [115]. Epigenetic regulation of immune cells in TME may also confer potential therapeutic implications for hematologic malignancies. Applying CITE-seq, M. Salmon et al. disclosed a new immunoregulatory mechanism in AML models treated with histone deacetylase inhibitor (HDACi) Panobinostat [116]. It was shown that plasmacytoid dendritic cells (pDCs) showed increased producing type I IFN with Panobinostat treatment by gaining H3K27ac near loci of the IFNα. Furthermore, combined treatment with the administration of Panobinostat and IFNα addressed the issue of pDCs depletion and led to improved clinical outcomes [116].

In addition to immune cells in the TME, the role of endothelial remodeling is also critically important for therapeutic targeting. For example, one recent study found that endothelial cells (ECs) can provide protumorigenic signals and sustain T-ALL cells during multiple drug treatments by reciprocally modulating their transcriptomic profile [117]. ScRNA-seq data implied T-ALL cells acquire “EC-mediated educational signature” including upregulation of JAK-STAT, MAPK, EGFR and TGF-β pathway and downregulation of p53 pathway, whereas ECs acquire more tumor-associating features including activation of VEGF-A, TNF-α, and NF-κB pathways.

Taken together, by providing insights at the single-cell level, these single-cell technologies enhance our understanding of drug resistance mechanisms mediated by TME and help identify new therapeutic targets.

### Deciphering drug effect and resistance by decoding therapy-induced clonal expansion and evolution

Understanding the alterations that occur within the leukemia composition after therapy was of vital importance, especially at the cellular level. The clonal expansion and selection, genetic mutation, and accumulation are inherently linked to resistance, owing to the selective pressure exerted by therapy on the resilient subpopulation [39]. A recent series of studies have utilized single-cell sequencing technology to explore the mechanisms of drug resistance caused by therapy-induced clonal expansion and evolution of tumor cells. These findings have been summarized in Table 6.

In AML, where the hierarchical structure is well established based on DNA sequence, Stentson et al. were the first to unveil the RNA-based clonal evolution of AML after therapeutic intervention [118]. Performing scRNA-seq on AML leukemia-initiating cells (LICs) from matched diagnosis and relapse bone marrow samples, the authors identified the common evolved gene expression and signaling networks mediating metabolism, apoptosis, and chemokine secretion in AML progression. Co-targeting CXCR4 and BCL2 marked increased survival in murine models [118]. Another study performing targeted scDNA-seq in CLL patients showed that the use of single-targeting agents, such as BTK inhibitors and BCL2 inhibitors, could give rise to a state of mutual exclusivity among resistance-associated genes in subclones of CLL patients [119]. Furthermore, the co-occurrence of multiple resistant mutations to different targeting agents can be also detected in the same clone [119].

Interrogating mtDNA-based clonal evolution is a novel method for decoding non-genetic mechanisms that contribute to relapse. By combining mtscATAC-seq, scRNA-seq and scATAC-seq, one study aimed to gain insights into CLL relapse by marking mtDNA mutations and chromosomal accessibility [120]. Mutations in mtDNA propagated more immensely under strong therapeutic pressure such as chemoimmunotherapy and allogeneic hematopoietic stem cell transplantation (allo-HSCT) compared to ibrutinib treatment. Paralleled to mtDNA mutation, chromosomal accessibility and expression profile also showed dynamics in CLL subclones. The depletion of transcription factor (e.g. SPIB, BCL11B, BCL11A, and IRF1) and an increase of CXCR4 expression in CLL was observed at relapse, indicating a less differentiated state [120].

In another trial focusing on CLL patients undergoing ibrutinib therapy with immunophenotyping, ATAC-seq, and scRNA-seq [121], robust reduced NF-κB binding activity was first observed after ibrutinib induction, followed by decreased regulatory activity of transcription factors involved in B cell development (e.g. EBF1, FOXM1, IRF4, PAX5, PU.1) and loss of B-cell surface markers in CLL cells (e.g. CD5 and CD19). Finally the acquisition of a quiescence-like gene signature was marked [121]. This study described the regulatory effects for therapeutic inhibition of B cell receptor signaling in CLL.

One study developed a linear-tracking system (ClonMapper), which utilized DNA barcoding in conjunction with CROP-seq [122], which is an expression vector for single-guide RNA (sgRNA) capable of expressing and capturing sgRNA barcodes in scRNA-seq [123]. This innovative approach enabled direct assessment of diversification and transcriptional patterns of clones [123]. Applying the tool to human CLL cell line system, the authors identified distinct pre-existing cell populations in the samples prior to treatment. One population comprised a unique subset of clones characterized by their noteworthy potential for “high survivorship” (HS). Following treatment, this subset expanded and accounted for the majority of relapse clones. The other population consisted of a subset of clones with a propensity for “low survivorship” (LS), which diminished after the therapeutic bottleneck. During the initial stages of treatment with fludarabine/mafosfamide, the HS subpopulation relies upon oxidative stress and DNA repair pathways to sustain their survival, whereas the LS subpopulation manifests mechanisms such as cellular senescence, inflammation, and translational control to alleviate cellular damage. These results proposed ClonMapper as a potent method in murine and humans for dissecting clonal dynamics involved in both tumor progression and the response to therapeutic interventions.

Allo-HSCT has been proven to have curative effects in treating hematologic malignancies with donor-derived graft-versus-leukemia (GvL) effect [124]. However, disease recurrence remains a significant challenge that restricts therapeutic efficiency [120, 125]. Single-cell transcriptomic together with epigenomic analysis revealed that the early relapse in CLL patients after allo-HSCT therapy is characterized by pre-existing stem-cell-like subpopulations. These subpopulations confer drug resistance, a mechanism that has also been observed in AML and CML through single-cell sequencing [15, 102, 103, 105]. Comparably, the late relapse (> 2 years) trajectory in CLL patients after allo-HSCT therapy exhibits divergent evolutionary paths and the gain of a broad range of methylome instability [125].

Glucocorticoids (GCs), functioning through the activation of pro-apoptotic pathways, were known as cell-growth inhibitors and were used for treating ALL for decades [126]. Poor primary response to GCs was often related to bad outcomes and relapse. In a single-cell study involving ALL patients treated with prednisolone (a kind of GC), a high risk of relapse was associated with activation of glucocorticoid response, smaller cell size, and a quiescent gene expression program with stemness properties (e.g. CD44, EPC1, SET2D, and SOCS2) [127]. Another recent study went deep into the mechanism of GC resistance and identified glucocorticoid receptor pathway was coordinated with the B-cell receptor (BCR) pathway in B-cell precursor acute lymphoblastic leukemia (BCP-ALL) [128]. By single-cell proteomics and RNA-seq, the authors identified that the BCR signaling pathway was enriched during GC treatment, with activation of BCR downstream pathway including the PI3K/mTOR and CREB signaling and accounted for GC resistance. Dasatinib effectively targeted these pathways and eliminated the resistance of GCs in vivo and in vitro [128]. This study suggested the combination of GCs and TKIs may potentially improve therapeutic outcomes in B-ALL patients.

Venetoclax (VEN) is a selective inhibitor of the anti-apoptotic protein BCL2, which has been associated with decreased sensitivity to chemotherapy [129]. Initially approved by the FDA in 2016 for the treatment of CLL with chromosomal 17p deletion, VEN has limited efficacy in treating AML as a monotherapy. However, the combination of VEN with DNA methyltransferase inhibitors or low-dose cytarabine in older patients, approved by the FDA, has shown promising results [131, 132]. Despite this, primary resistance and adaptive resistance through clonal selection can lead to chemotherapy-refractory relapse [133]. Recent studies utilizing single-cell sequencing have helped decipher the complex clonal evolutionary nature of leukemia with VEN-based treatment [133–136].

One study identified that primary and adaptive resistance to VEN-based therapy correlated with the acquisition or enrichment of different kinase-activating clones in AML, such as FLT3-ITD, FLT3-TKD, FLT3 N676K, and RAS mutations, whereas FLT3-ITD gain and TP53 loss were considered to account for the VEN resistance [133]. Another study taking advantage of scDNA-seq revealed that the adaptive subclone to VEN + Ara-c treatment exhibited changes in oxidative phosphorylation, ETC complex I, and the TP53 pathway [134]. Subsequent trials showed the treatment of ETC inhibitors, pyruvate dehydrogenase inhibitors, or mitochondrial ClpP protease agonists largely postponed the relapse following VEN + Ara-c treatment, promoting new potential therapeutic targets related to metabolism in AML [134]. Furthermore, a study that profiled DNA mutations, methylation patterns, metabolism, and expression identified and validated the RAS/MAPK pathway-induced MCL-1 expression as an acquired pathway of VEN resistance [135]. scDNA-seq revealed the clonal selection in AML patients treated with VEN, showing the clear clonal expansion of clones harboring RAS mutation [135]. These findings established the potential combinatorial treatment strategy related to the RAS/MAPK/MCL-1 pathway [135]. The upregulated ubiquitination of MCL-1 has also been observed in CLL patients who relapsed with VEN monotherapy, which may be directly associated with NF-κB activation [136]. With the recent clinical trial combining VEN + DA treatment achieving a 91% remission rate in AML patients [137], there is growing interest in expanding VEN-based regimens for effective treatment. These studies establish the ample scope for single-cell analysis on VEN-based therapy and identified several new potential targets for therapy and prognosis in leukemia.

## Perspectives

### Towards spatial dissection of leukemia at single-cell level

Single-cell omics methodologies, such as spatial omics [138], scCUT&tag [139], scHi-C [24] and multi-omics, are continuously expanding their repertoire, encompassing additional cellular dimensions. For example, the pathology of leukemia always occurs in a spatial context, disseminating from the bone marrow and establishing tumor niches in various environments, including the central nervous system [140]. State-of-art spatial transcriptomes are available by laser capture microdissection (LCM)-based strategies and image-based strategies, enabling in situ and high-resolution spatial transcriptome profiling of single cells in the TME [138]. Recently, barcoding-based spatial transcriptomics by DBiT-seq has reduced costs and allowed for the quantification of spatial epigenomics [141], opening the way for spatial multiomics and may reveal the spatial regulatory networks. Proteins, the functional units in the cell, can now be quantified in spatial proteomics at near single-cell level, thanks to recent advances in liquid chromatography-mass spectrometry (LC-MS) based methods and matrix-assisted laser desorption/ionization (MALDI) [142]. Isolation and dissection of the spatially diversely organized spatial structure of leukemia TME may uncover the spatial programming of the small niches and drug resistance mechanisms [143].

### Deep into single-cell data by artificial intelligence

Considering the burgeoning expansion of single-cell omics data, the concurrent computational data processing and interpretation methods to comprehend this wealth of information hold equal significance. Biomedical research endeavors are increasingly employing artificial intelligence (AI), specifically deep learning (DL), to enhance the dependability of analytical workflows and discern latent molecular characteristics. DL frameworks have been devised to discriminate between molecular subtypes in various cancers [144]. In the context of leukemia, different DL framework (AMLnet [145], CMLcGAN [146], ALNett [147]) has been applied to the diagnosis and classification of leukemia from medical images. The rapid emergence of AI that integrate and analyze omics data is happening in parallel with advancements in single-cell technologies. For example, one study implemented machine learning for AML cell-type differentiation. By defining a hierarchy framework along the HSC to the myeloid axis, they successfully classified AML cells into six subclasses, providing huge insight into AML heterogeneity [58].

However, studies combining AI/DL with single-cell omics datasets to identify distinct subtypes and predict interactions in TME and drug response are still lacking in leukemia. With the ongoing generation of data derived from diverse single-cell omics of leukemia samples, the amalgamation of multi-omics data with AI-based analytical approaches holds great promise in making significant strides toward understanding the etiology, drug resistance mechanisms, discovery of novel targets, and prognostication on leukemia in the future.

### Leveraging single-cell technologies for developing precision medicine against leukemia

Precision medicine in leukemia seeks to enhance patient outcomes by customizing treatment based on the distinctive genomic characteristics of the tumor. Previously, large-scale genomic projects such as The Cancer Genome Atlas [148] (TCGA) have built a roadmap to genetic changes present in various cancer subtypes before the commencement of treatment. However, bulk omic data is still limited in giving precise insights into intra-tumor heterogeneity as the profile was averaged. As largely reviewed above, single-cell sequencing has the intrinsic advantage of tracking personal tumor traits. For example, analyzing the evolutionary structure of leukemia by single-cell sequencing could address how and at what stage the tumor has progressed, aiding the fine-tuning of effective personal therapeutic strategies. One study has successfully realized the prediction of AML drug response based on the sequencing result in mouse patient-derived xenografts (PDX) model [59]. This paves the way for tailored treatment strategies in patients, indicating a new era in personalized precision medicine of leukemia. Besides, examining the personalized composition and repertoire of tumor-infiltrating immune cells by single-cell sequencing is also essential as it is directly linked to the efficiency of immune checkpoint blockade.

However, it is worth noting that we still are on the way to incorporating single-cell technologies into clinical practice. Issues such as the absence of a comprehensive tumor-associated single-cell sequencing database, the sparsity in single-cell data, data bias from different experimental batches and studies, and the relatively high cost of sequencing are still posing challenges [149]. With the rapid advance of sequencing techniques and cost reduction, we firmly believe that the clinical implementation of single-cell technologies would be one of the most important strides toward precision medicine of leukemia and other cancers in the near future.

### Electronic supplementary material

Below is the link to the electronic supplementary material.

**Supplementary Material 1: Supplementary Table S1.** The list of abbreviations
